# BAFF 60-mer, and Differential BAFF 60-mer Dissociating Activities in Human Serum, Cord Blood and Cerebrospinal Fluid

**DOI:** 10.3389/fcell.2020.577662

**Published:** 2020-11-06

**Authors:** Mahya Eslami, Edgar Meinl, Hermann Eibel, Laure Willen, Olivier Donzé, Ottmar Distl, Holm Schneider, Daniel E. Speiser, Dimitrios Tsiantoulas, Özkan Yalkinoglu, Eileen Samy, Pascal Schneider

**Affiliations:** ^1^Department of Biochemistry, University of Lausanne, Epalinges, Switzerland; ^2^Institute of Clinical Neuroimmunology, University Hospital of the Ludwig-Maximilians-Universität München, Munich, Germany; ^3^Faculty of Medicine, Center for Chronic Immunodeficiency, Medical Center – University of Freiburg, Freiburg, Germany; ^4^AdipoGen Life Sciences, Epalinges, Switzerland; ^5^Institute for Animal Breeding and Genetics, University of Veterinary Medicine Hannover, Hannover, Germany; ^6^Department of Pediatrics, Friedrich-Alexander University Erlangen-Nürnberg, Erlangen, Germany; ^7^Department of Oncology, University of Lausanne, Lausanne, Switzerland; ^8^Department of Laboratory Medicine, Medical University of Vienna, Vienna, Austria; ^9^Clinical Pharmacology, Quantitative Pharmacology, Translational Medicine, Merck KGaA, Darmstadt, Germany; ^10^Business of Merck KGaA, EMD Serono Research & Development Institute, Inc., Billerica, MA, United States

**Keywords:** B-cell activating factor, cerebrospinal fluid, serum, cord blood, 60-mer, atacicept, belimumab

## Abstract

B cell activation factor of the TNF family (BAFF/BLyS), an essential B cell survival factor of which circulating levels are elevated in several autoimmune disorders, is targeted in the clinic for the treatment of systemic lupus erythematosus (SLE). The soluble form of BAFF can exist as 3-mer, or as 60-mer that results from the ordered assembly of twenty 3-mers and that can be obtained from naturally cleaved membrane-bound BAFF or made as a recombinant protein. However, which forms of soluble BAFF exist and act in humans is unclear. In this study, BAFF 3-mer and 60-mer in biological fluids were characterized for size, activity and response to specific stimulators or inhibitors of BAFF. Human cerebrospinal fluids (CSF) from patients with multiple sclerosis and adult human sera contained exclusively BAFF 3-mer in these assays, also when BAFF concentrations were moderately SLE or highly (BAFFR-deficient individual) increased. Human sera, but not CSF, contained a high molecular weight, saturable activity that dissociated preformed recombinant BAFF 60-mer into 3-mer. This activity was lower in cord blood. Cord blood displayed BAFF levels 10-fold higher than in adults and consistently contained a fair proportion of active high molecular weight BAFF able to dissociate into 3-mer but not endowed with all properties of recombinant BAFF 60-mer. If BAFF 60-mer is produced in humans, it is dissociated, or at least attenuated in the circulation.

## Introduction

B cell activating factor (BAFF), a member of TNF family ligands, is a factor for the survival and development of B cells, as evidenced by the sharp reduction of peripheral B cells in BAFF-deficient mice (Schiemann et al., 2001; Craxton et al., 2005; Mackay and Schneider, 2009). Like other TNF family ligands, BAFF is a type II membrane-bound protein. It is expressed by cell types like macrophages, dendritic cells, neutrophils and monocytes, but also by stromal cells like astrocytes or carcinoma cells (Mackay et al., 2003; Krumbholz et al., 2005; Kato et al., 2006; Giordano et al., 2020). BAFF can be proteolytically processed by furin to release a soluble trimeric ligand, or can remain membrane-bound (Craxton et al., 2003; Bossen and Schneider, 2006). BAFF binds to three different receptors: BAFFR (BAFF receptor), TACI (transmembrane activator and CAML interactor) and BCMA (B cell maturation antigen), which are expressed on B lineage cells at different stages of their development (Bossen and Schneider, 2006). BAFF can form biologically active heteromers with A proliferation inducing ligand (APRIL), a related member of the TNF family (Hahne et al., 1998). Heteromers were first detected in the serum of patients with rheumatic diseases (Roschke et al., 2002). APRIL and BAFF-APRIL heteromers share with BAFF the two receptors TACI and BCMA (Schuepbach-Mallepell et al., 2015). BAFF activates non-canonical and/or canonical NF-κB pathways (Claudio et al., 2002; Hatada et al., 2003), which upregulate anti-apoptotic factors like Mcl-1 to improve B lymphocyte survival [reviewed in Mackay and Schneider (2009)]. Similar to the TNF system, in which soluble TNF is the prime activating ligand for TNFR1 while membrane-bound TNF more specifically stimulates TNFR2 (Grell et al., 1995), BAFF and APRIL receptors may respond differently to various forms of ligands. *In vitro* data indicate that, unlike BAFFR, TACI does not respond to the action of trimeric BAFF or APRIL, but requires higher order oligomers of these ligands to become activated efficiently (Bossen et al., 2008). These oligomers may mimic the action of membrane-bound ligands.

Circulating BAFF levels are elevated in patients with systemic lupus erythematosus (SLE; Zhang et al., 2001; McCarthy et al., 2013; Salazar-Camarena et al., 2016), multiple sclerosis (MS; Kannel et al., 2015; Steri et al., 2017), rheumatoid arthritis (Cheema et al., 2001), or IgA nephropathy (Xin et al., 2013; Li et al., 2014). A genetic variant of BAFF, enriched in Sardinia, results in elevated serum levels of BAFF and is associated with a risk for MS (Steri et al., 2017). Outside of Sardinia, serum levels of BAFF were found to be elevated in some (Kannel et al., 2015), but not all (Krumbholz et al., 2008) studies, but were consistently found to be elevated in response to IFN-β therapy (Krumbholz et al., 2008; Kannel et al., 2015) and rituximab (Pellkofer et al., 2008). Additionally, genetic alterations in BAFFR or TACI genes can lead to common variable immunodeficiency (CVID) which is characterized by hypogammaglobulinemia and recurrent respiratory or intestinal tract infections (Rosen et al., 1999; Warnatz et al., 2009). Individuals with BAFFR deficiency show defective B cell development and lower level of IgM and IgG. In contrast, circulating levels of BAFF are higher than in controls by one to two orders of magnitude (Warnatz et al., 2009; Kreuzaler et al., 2012). All receptors for BAFF and APRIL can be processed to soluble forms (Hoffmann et al., 2015; Laurent et al., 2015; Smulski et al., 2017). Soluble TACI and BCMA were present and shown to act as decoy receptors in SLE patients, with the result of blocking NF-κB signaling and subsequent B cell survival, at least *in vitro* (Hoffmann et al., 2015; Laurent et al., 2015). BAFF antagonists are investigated in the clinic to prevent activation of B cell-driven mechanisms that contribute to the pathology of autoimmune diseases. Belimumab (trade name Benlysta) is a human monoclonal antibody against human BAFF which has been approved for the treatment of lupus in 2011 (Hahn, 2013). Atacicept is a fully human recombinant protein in which the ligand-binding portion of the extracellular domain of TACI is fused to the Fc portion of a human IgG1 engineered not to bind Fc receptors and complement. Atacicept significantly decreased circulating B cells and antibodies in treated individuals and showed promising efficacy results in a phase IIb clinical trial on patients with active, autoantibody-positive SLE, under standard therapy (Merrill et al., 2018). Belimumab and atacicept both inhibit membrane-bound and soluble BAFF, but differ in their target specificity with regards to APRIL, BAFF-APRIL heteromers and BAFF 60-mer which are inhibited by atacicept but not by belimumab (Schuepbach-Mallepell et al., 2015; Kowalczyk-Quintas et al., 2018). BAFF 60-mer is an unusual form for a TNF family ligand in which twenty 3-mer are ordered in a pH-dependent capsid-like structure. It was discovered in 2002, when recombinant BAFF was crystallized alone or in complex with BAFFR or BCMA (Liu et al., 2002, 2003). Initial concerns that pH-dependent 60-mer formation might be an artifact of the poly-histidine tag used for purification (Zhukovsky et al., 2004) were wiped by the demonstration that untagged BAFF produced in yeast also formed 60-mer, with pH dependence being explained by the important role of a histidine residue (H218; Cachero et al., 2006). H218 is located in a unique loop of BAFF involved in BAFF-BAFF interactions and that serves two functions. The first is to allow weak and transient 3-mer to 3-mer interactions, that have no effect on receptor binding but are essential to induce productive signaling through BAFFR, probably by allowing interactions of BAFF-BAFFR complexes once BAFF has bound to receptors. This function characterized both *in vitro* and *in vivo* does not require 60-mer formation as it is not affected by mutation H218A, but is destroyed by the more “severe” E223K mutation in the flap (Vigolo et al., 2018). The second function is the formation and stabilization of BAFF 60-mer, in which each of the twenty BAFF 3-mer interacts with 3 neighbors via flap-flap interactions crucially involving His218 (Liu et al., 2002; Cachero et al., 2006; Vigolo et al., 2018). Cross-linking of BAFF with antibodies that do not interfere with receptor binding not only rescues the activity of “flap-dead” BAFF mutants, but also stimulates the activity of wild type BAFF (Kowalczyk-Quintas et al., 2016; Vigolo et al., 2018). Transition of BAFF 60-mer to BAFF 3-mer at pH ≤ 7 is believed to rely on protonation of His218. Atacicept can inhibit BAFF 60-mer, but belimumab cannot because its binding epitope in BAFF 60-mer is inaccessible for steric hindrance reasons (Shin et al., 2018; Vigolo et al., 2018). Given (a) the superior activity of BAFF 60-mer over 3-mer (Liu et al., 2002, 2003), (b) its potential to stimulate receptors that BAFF 3-mer cannot (Bossen et al., 2008), (c) its differential susceptibility to clinical BAFF antagonists (Shin et al., 2018; Vigolo et al., 2018), and (d) the complete absence of data regarding its occurrence in humans, we characterized BAFF in human serum and other biological fluids making use of five criteria that are specific for BAFF 60-mer: its size, its high activity, its pH-sensitivity, its refractoriness to inhibition by belimumab and the inability to further activate its activity with cross-linking anti-BAFF antibodies. In this study, we distinguished three types of biological fluids: (i) human serum that had no or very little detectable endogenous BAFF 60-mer. On the contrary, a BAFF 60-mer inhibitory activity able to dissociate spiked recombinant 60-mer into 3-mer was present in adult human sera. Human lymph exudates behaved similarly. (ii) cerebrospinal fluid (CSF) that contained neither BAFF 60-mer nor BAFF 60-mer inhibitory activity and (iii) cord blood samples that contained low levels of inhibitory activity but all displayed a fair proportion of active, high molecular weight BAFF with the size of BAFF 60-mer. Similar to BAFF 60-mer, the specific activity of high molecular weight BAFF was higher than that of BAFF 3-mer. Also, like BAFF 60-mer, high molecular weight BAFF could dissociate into 3-mer. However, high molecular weight BAFF was recognized and inhibited by antibodies unable to bind undissociated recombinant BAFF 60-mer, suggesting either that high molecular weight BAFF is not a 60-mer, or that it is an easy-to-dissociate BAFF 60-mer. Regarding the BAFF 60-mer dissociating activity, it had a high molecular weight, was resistant to protease inhibitors and to heating at 56°C, did not bind to immobilized BAFF but was inactivated by boiling. We also describe that endogenous BAFF 3-mer does not re-associate as 60-mer, even under favorable conditions after affinity purification. Our data suggest two possible scenarios. In the first one, BAFF 60-mer does not exist *in vivo* and high molecular weight BAFF present in cord blood is part of an undefined complex. In the second one, BAFF 60-mer can form locally but is actively dissociated in adult human serum. It can persist in cord blood, but in a more labile form than recombinant BAFF 60-mer.

## Materials and Methods

### Human and Animal Samples

Normal adult human serum samples and cord blood samples were as described (Podzus et al., 2017). Matched pairs of serum and plasma were collected under the approval of the Ethics Committee of the Medical University of Vienna, Austria (EK Nr: 1845/2015). Human SLE serum samples were from patients who were enrolled in the randomized, double-blind, APRIL-SLE trial, but before they received any treatment with atacicept (ClinicalTrials.gov Identifier NCT00624338). Serum sample from a BAFFR-deficient person, of a Bruton’s tyrosine kinase (BTK)-deficient patient and a CVID patient were as previously described (Warnatz et al., 2009; Kreuzaler et al., 2012). CSF samples from MS patients were provided by the Institute of Clinical Neuroimmunology, Munich. This was approved by the Ethical Committee of the Medical Faculty of Ludwig-Maximilians-Universität München. Human lymphatic exudate samples were collected from three melanoma patients after sentinel lymph node surgery. Lymph was centrifuged and stored at −20°C until use (Broggi et al., 2019). For cows, sera were from purebred German Fleckvieh, Vorderwald, German Holstein cattle and from a Vorderwald by German Holstein crossbred. All animal work was conducted according to national and international guidelines for animal welfare. The Lower Saxony state veterinary office at the Niedersächsisches Landesamt für Verbraucherschutz und Lebensmittelsicherheit, Oldenburg, Germany, was the responsible Institutional Animal Care and Use Committee (IACUC) for this study. This specific study had been approved by the IACUC of Lower Saxony, the state veterinary office Niedersächsisches Landesamt für Verbraucherschutz und Lebensmittelsicherheit, Oldenburg, Germany (registration number 33.42502-05-04A247). Mouse sera were obtained by puncture of the facial vein of C57Bl6 mice according to Swiss Federal Veterinary Office guidelines, and under the authorization of the Office Vétérinaire Cantonal du Canton de Vaud (authorization 1370.7 to PS). Blood was incubated for 2 h at 37°C, spun at 13,000 rpm for 15 min at 4°C and supernatant was collected.

### Proteins and Antibodies

Belimumab (registered trade name Benlysta) and etanercept (TNFR2-Fc, registered trade name Enbrel) were bought from the Pharmacy of Lausanne University Hospital (CHUV). Rat IgG2b anti-human BAFF monoclonal antibody 2.81 (Kreuzaler et al., 2012) was from Adipogen (#AG-20B-0018-C100). Mouse IgG anti-APRIL monoclonal antibody 104 was co-developed with and provided by Adipogen. Its characterization will be described in detail elsewhere. Mouse IgG1 anti-SHH 5E1 (Wang et al., 2000) was purified from hybridoma supernatants obtained from Developmental Studies Hybridoma Bank (University of Iowa, Department of Biology, Iowa City, IA, United States). Rat IgM anti-human BAFF monoclonal antibody Buffy2 was as described (Schneider et al., 1999). Atacicept was provided by Merck KGaA. Fc-BAFF and BCMA-Fc were stably transfected and produced in CHO cells and affinity-purified on Protein A-Sepharose as previously described (Schneider, 2000), or were from Adipogen [Fc-BAFF, AG-40B-0120 and BCMA(h):Fc(h), AG-40B-0080]. Fc-BAFF, atacicept, belimumab, mAb 104, and mAb 5E1 were coupled at 2 (Fc-BAFF, 104, 5E1) or 5 mg/ml (atacicept, belimumab, etanercept) to N-hydroxysuccinimide (NHS)-Sepharose beads (GE Healthcare #90-1004-00) according to manufacturer’s instructions. An expression plasmid for Flag-BAFF was transiently transfected in 293T cells with the polyethyleneimide method (Tom et al., 2008). 7 days later, 400 ml of conditioned supernatants in serum-free OptiMEM medium were purified on a 1 ml column of atacicept-coupled Sepharose, eluted with 50 mM citrate-NaOH pH 2.7, neutralized with 1 M Tris–HCl pH 9, and buffer was exchanged for PBS by ultrafiltration in a centrifugal device with 30 kDa cut off (Amicon Ultra-4, Merck Millipore, #10210342). Flag BAFF forms exclusively 3-mer. It was not further purified by size exclusion chromatography (SEC). It was quantified by absorbance at 280 nm using an extinction coefficient of 16055 M^–1^ cm^–1^ (absorbance at 1 mg/ml of 0.866). Naturally cleaved BAFF (wt, H218A or E223K) in about 15 ml of conditioned cell supernatants of transfected 293T cells was affinity purified on 12 μl of atacicept-coupled Sepharose beads and size fractionated by SEC in 20 mM Hepes, 130 mM NaCl, 10 μg/ml BSA, pH 8.2. Fractions corresponding to BAFF 60-mer (8–10 ml) and BAFF 3-mer (14–16 ml) were pooled, aliquoted and stored at −70°C until use. Naturally cleaved BAFF 60-mer and 3-mer were quantified by BAFF ELISA with a capture step at pH 5.5 (see section “ELISA”). 3-mer fractions of BAFF mutants H218A and E223K were quantified by Western blot using purified His-BAFF 60-mer as a standard and mAb Buffy2 to reveal. His-BAFF 60-mer expressed in Escherichia *coli* was from Adipogen (AG-40B-0112-C010). All plasmids used in this study are listed in Supplementary Table 1.

### Cell Lines

HEK 293T cells were obtained from late Jürg Tschopp (University of Lausanne) and grown in DMEM 10% FCS. Jurkat JOM2 BAFFR:Fas-2308 cl21 and Jurkat BCMA:Fas-2309 clone 13 reporter cells were described previously and were grown in RPMI 10% FCS (Bossen et al., 2008; Nys et al., 2013; Schneider et al., 2014; Schuepbach-Mallepell et al., 2015). CHO-S cells were from Thermoscientific (A1155701). CHO-S-2825 clone G5 expressing Fc-BAFF was obtained by transfection of CHO-S cells by the polyethyleneimide method, selection by 3 passages in 500 μg/ml of G418 sulfate (Calbiochem, 345812) and cloning by limiting dilution. The clone with highest production as assessed by Western blot with horseradish peroxidase-coupled goat anti-human Fc antibodies was selected for production.

### Cytotoxic Assay

The activity of endogenous or recombinant BAFF was measured using Jurkat BCMA:Fas-2309 clone 13 or Jurkat JOM2 BAFFR:Fas-2308 clone 21 reporter cells (Schneider et al., 2014). In flat-bottomed 96 well cell culture plates, samples were serially diluted as indicated into a final volume of 50 μl of RPMI, 10% FCS. Then, 50 μl of reporter cells (20’000–50’000/well) in the same medium were added and incubated overnight (∼16 h) at 37°C, 5% CO_2_, after which time cell viability was monitored by addition of 20 μl of PMS/MTS (phenazine methosulphate at 45 μg/ml and 3-(4,5-dimethylthiazol-2-yl)-5-(3-carboxymethoxyphenyl)-2-(4-sulfophenyl)-2H-tetrazolium at 2 mg/ml in PBS) and measuring absorbance at 492 nm after 2–8 h (Nys et al., 2013; Schneider et al., 2014; Schuepbach-Mallepell et al., 2015). When tests were performed in the presence of modifiers of BAFF activity (atacicept, belimumab, or anti-BAFF 2.81), modifiers at 10-fold the desired final concentration in 10 μl of RPMI 10% FCS were added, followed by reporter cells in a volume of 40 μl instead of 50 μl. When tests were performed to measure the inhibitory activity of serum or other biological fluids on recombinant BAFF 60-mer, 2 μl of sera or fluid were added per well, unless stated otherwise. In some instances, serum was heated for 30 min at 56°C. In other instances, size exclusion chromatography fractions of normal human serum were heated for 5 min at 95°C, then spun for 15 min at 13,000 rpm in a tabletop centrifuge to remove precipitated proteins, and supernatant were used in the assay. Where indicated, one-fold concentrated protease inhibitor cocktail (Sigma, “cØmplete,” 11697498001) was added to serum prior to the assay. Reporter cells were not affected by this concentration of protease inhibitors in the time frame of the assay. Optionally, antibiotics (Invitrogen, 15070–063) were added in samples or cells to have a final concentration of 50 U/ml streptomycin and 50 μg/ml penicillin, in particular when non-sterile samples were tested, such as size exclusion chromatography fractions. For the estimation of the percentage of high molecular weight BAFF at the activity level after size exclusion chromatography, EC_50_ expressed in μl of fraction was first determined for fractions 9, 14, and 15, then the following calculation was performed: % high molecular weight BAFF activity = [(1/EC_50_ of fraction 9)/(sum of (1/EC_50_) of fractions 9, 14, and 15)] × 100.

### BAFF ELISA

Endogenous or recombinant human BAFF was quantified using BAFF (human) ELISA kit from Adipogen (#AG-45B-0001-KI01) according to the manufacturer’s protocol, using 2.5 μl or 10 μl of human sera as indicated, or 3 μl of serum from cord blood, or 100 μl of human CSF samples. For SEC fractions, adjusted volumes were used for the BAFF ELISA (Supplementary Table 2). The capture step was performed in ELISA buffer provided with the kit (pH 7.4). When indicated, for the detection of BAFF 60-mer, the capture step was performed for 3 h at room temperature in MES [2-(N-morpholino)ethanesulfonic acid] buffer pH 5.5. For this purpose, suitable amounts of 0.5 M MES pH 5 were added to samples prior to the capture step of the ELISA. This amount was determined for each type of buffer by controlling pH on a pH paper with a 0.5 pH unit scale. For the measurement of endogenous BAFF in 200 μl cord blood right after size exclusion chromatography, 150 μl of 1 ml fractions were immediately captured for 30 min at 4°C and pH 7.4 or pH 5.5. For the estimation of the percentage of high molecular weight BAFF at the protein level, the following calculation was performed: % high molecular weight BAFF protein = [signal in fraction 9/(sum of signals in fractions 9, 14, and 15)] × 100.

### Size-Exclusion Chromatography

A dedicated Superdex S200 Increase HR 10/30 columns was used for the analysis of samples containing endogenous BAFF, and another for samples containing recombinant BAFF. This can explain small differences in the retention time of standards. Size-exclusion chromatography with 200 to 400 μl of samples was performed at a flow rate of 0.65 ml/min in 20 mM Hepes, 130 mM NaCl, pH 8.2. For diluted samples in the absence of a protein matrix, 10 μg/ml bovine serum albumin was added in the buffer. For samples with low endogenous BAFF levels requiring subsequent lyophilization, 10 mM Hepes, 30 mM NaCl, 10 μg/ml BSA, pH 8.2 was used. Fractions of 1 ml were collected. Lyophilized fractions were suspended into 100 or 200 μl of water to get 10-fold or 5-fold concentrated fractions, including salts and buffer. When indicated, pooled fractions were concentrated using 30 kDa cut off centrifugal concentration devices to a volume of about 300 μl prior to re-injection. Columns were calibrated with 100 μl of a mixture of protein standards, all at 1.4 mg/ml (except ferritin at 140 μg/ml): thyroglobulin (669 kDa), ferritin (440 kDa), aldolase (158 kDa), ribonuclease A (13.7 kDa; all from GE Healthcare), bovine serum albumin (67 kDa), ovalbumin (43 kDa), carbonic anhydrase (29 kDa), and aprotinin (6.5 kDa; all from Sigma-Aldrich).

### Immunoprecipitation

To purify or deplete endogenous or recombinant BAFF from human serum, CSF or other samples, samples were mixed with 20 μl of a 50% slurry in PBS of NHS-Sepharose beads coupled to the desired protein or antibody, and incubated overnight at 4°C on a rotating wheel. Beads were centrifuged for 5 min at 5,000 rpm (2,400 × *g*). The unbound fraction was collected, while beads were washed 3 times with 100 μl of PBS in mini columns (Schneider et al., 2014) and eluted with 30 μl of 50 mM citrate-NaOH pH 2.7. The eluate was neutralized with 10 μl of 1 M Tris–HCl pH 9.

### Western Blot

Sodium dodecyl sulfate polyacrylamide gel electrophoresis (SDS-PAGE) of 12% acrylamide gels and Western blot on nitrocellulose membranes were performed according to standard protocols. His-BAFF-60mer at 50, 25, 12.5, and 6.25 ng per lane was used as a standard. Membrane were revealed with Buffy2 at 1 μg/ml, followed by horse radish peroxidase-coupled goat anti-rat IgM, μ chain specific (Jackson ImmunoResearch, 112-035-075) at 1/8000 and ECL. Concentrations of naturally cleaved BAFF H218A and E223K were estimated by comparing band intensities. The same Western blot procedure was used to reveal naturally cleaved BAFF in fractions of size exclusion chromatographies.

### Statistics

Statistics were performed with Prism 8 (GraphPad Software). Normal distribution of data was assessed with D’Agostino Pearson normality test for *n* ≥ 8, or assumed to be so for *n* < 8. Standard deviations were not assumed to be equal and comparisons of multiple groups was performed by Brown-Forsythe and Welch ANOVA test, followed by Dunnett T3 multiple comparison tests. For the comparison of 2 groups, *t*-test with Welch’s correction was used. Differences were considered significant when *P* < 0.05. To determine the EC_50_ of titration curves, cell viability was first normalized, then fitted with the “Non-linear regression (curve fit)” followed by the “log(agonist) vs. normalized response-variable slope” functions of Prism 8 (GraphPad Software).

## Results

### Elevated BAFF in BAFFR-Deficient Human Serum Is Exclusively in a Trimeric Form

**FIGURE 1:**
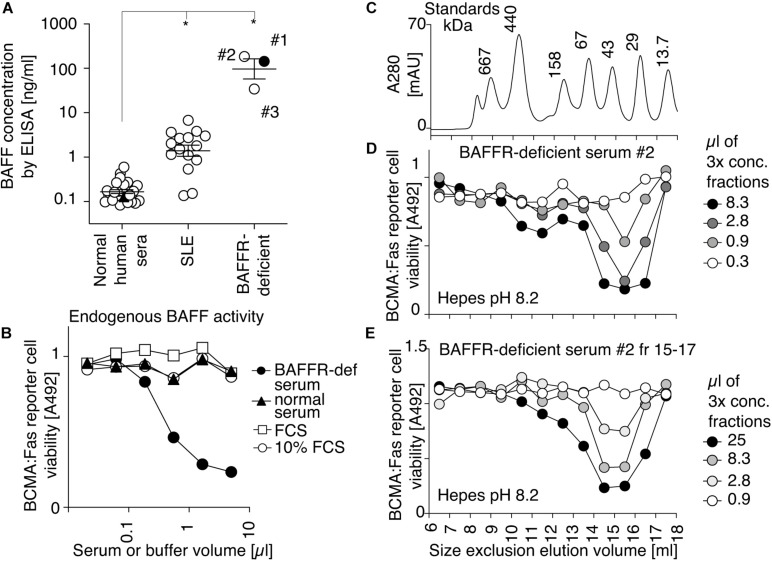
Endogenous BAFF activity in serum of a BAFFR-deficient person has the size of a 3-mer. **(A)** Concentrations of endogenous BAFF measured by ELISA in 2.5 μl of normal human sera, in sera of SLE patients and in three independent serum samples of a BAFFR-deficient person. * indicates significant (*P* < 0.001) difference with the control group of normal human sera by one-way ANOVA with Bonferroni’s multiple comparison test. One experiment out of three with comparable results is shown. **(B)** The activity of endogenous BAFF in the indicated volume of BAFFR-deficient and normal human serum, or in foetal calf serum (FCS), or in culture medium (10% FCS) was monitored with BCMA:Fas reporter cells. The final assay volume was 100 μl. Cell viability was measured with the PMS/MTS assay. This experiment was performed twice in this format, and three more times on similar reporter cells (BAFFR:Fas). **(C)** A mixture of molecular weight markers (top panel) was fractionated by SEC in Hepes buffer pH 8.2, and detected by on-line monitoring of absorbance at 280 nm. **(D)** a BAFFR-deficient human serum was fractionated by SEC in Hepes buffer pH 8.2. Fractions were concentrated 3-fold by ultrafiltration and BAFF activity in each fraction was monitored by its ability to kill BCMA:Fas reporter cells. The experiment was performed three times (plus once with a different readout). **(E)** Fractions 15–17 of the chromatography shown in panel **(D)** were pooled, concentrated by ultrafiltration, fractionated again by SEC, and analyzed for BAFF activity as shown in panel **(D)**. This experiment was performed once.

Serum samples collected at different times from a BAFFR-deficient individual displayed BAFF levels by ELISA that were on average 500-fold higher than those of controls and 50-fold higher than those of SLE patients (Figure 1A). BAFF in BAFFR-deficient serum, but not normal serum, was detectable in a cell-based activity assay, in which target cells are Jurkat T cells expressing the chimeric receptor BCMA:Fas (Figure 1B). These cells divert BAFF (and APRIL) signals into death via the intracellular domain of the apoptosis-inducing receptor Fas. Endogenous BAFF and APRIL in normal human serum were under the detection limit (Figure 1B). As APRIL levels are not elevated in BAFFR-deficient serum (unpublished observation), APRIL likely did not contribute to signal in this experiment, as will be confirmed later with BAFF-specific reporter cells. The human BAFFR-deficient serum was thus used to investigate the ratio of activity associated with BAFF 3-mer and BAFF oligomers after a size-fractionation performed at pH 8.2, a pH that is favorable to BAFF 60-mer (Cachero et al., 2006). BAFF activity was recovered in late fractions (15–17). No activity was detected in early fractions (9 and 10) that would correspond to BAFF 60-mer (Figures 1C,D). To test the hypothesis that BAFF assembly into 60-mer at pH 8.2 might be a slow process, fractions 15–17 were pooled, concentrated and size-fractionated again at pH 8.2, but BAFF activity still eluted in late fractions (Figure 1E). The theoretical molecular weight of naturally processed BAFF is 51 kDa (3 × 17 kDa), and calibration markers indicated an apparent size of 46 kDa for endogenous BAFF activity (2.7-mer). Under identical conditions, a recombinant His-BAFF that was undoubtedly trimeric by electron microscopy and crystallization also eluted as an apparent 2.7-mer relative to molecular weight markers (Vigolo et al., 2018). Taken together, these results indicate that endogenous BAFF in BAFFR-deficient serum is present as 3-mer, and that the absence of 60-mer is not a consequence of a potentially inadequate pH of serum.

### Human Serum Contains a High Molecular Weight Inhibitory Activity for BAFF 60-mer

**FIGURE 2:**
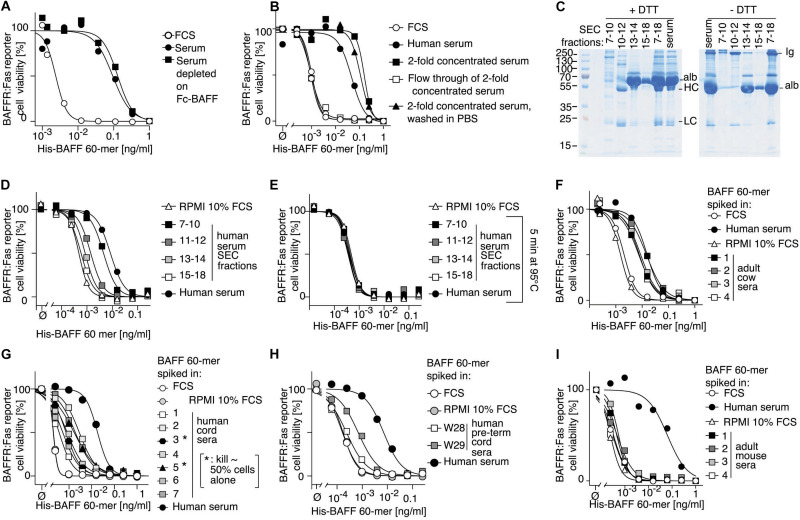
Human serum contains a BAFF 60-mer inhibitory activity. **(A)** Recombinant His-BAFF 60-mer was titrated on BAFFR:Fas reporter cells in the presence of a constant amount of FCS (10%), or of normal human serum, or of normal human serum depleted on Fc-BAFF-coupled Sepharose beads. After an overnight incubation, cell viability was monitored. The experiment was performed three times. **(B)** Normal human serum was concentrated 2-fold by ultrafiltration, then washed with PBS by ultrafiltration. FCS, normal serum, 2-fold concentrated normal serum before and after wash with PBS, and the filtrated fraction of normal serum were tested on BAFFR:Fas reporter cells as described in panel **(A)**. The experiment was performed four times. **(C)** 400 μl of normal serum was size-fractionated by SEC. The indicated fractions were pooled, concentrated to 400 μl, and 1 μl was analyzed by SDS-PAGE and Coomassie blue staining under reducing (+DTT) or non-reducing conditions (-DTT). This experiment was performed twice. **(D)** Serum fractions as shown in panel **(C)** were analyzed for their BAFF 60-mer inhibitory activity as described in panel **(A)**. The experiment was performed four times. **(E)** Same as panel **(D)**, except that supernatants of fractions heated for 5 min at 95°C were analyzed. The result with medium only is the same as in panel **(D)**. **(F–I)** four adult cow sera **(F)**, 7 sera from human cord blood **(G)**, two sera from cord blood of pre-term babies at gestational weeks 28 and 29 **(H)** and 4 adult mouse sera **(I)** were analyzed with the indicated controls as described in panel **(A)**. In panel **(G)**, 3* and 5* indicate that cord sera 3 and 5 contained an intrinsic BAFF activity that killed about 50% of reporter cells in the absence of BAFF 60-mer. Experiment **(E)** was performed once. Experiments **(F)**, **(G)**, and **(I)** were performed three times each, and experiment **(H)** was performed twice.

We wondered whether BAFF 60-mer activity would have been detected if present in serum. Thus, the activity of recombinant His-BAFF 60-mer (Vigolo et al., 2018) spiked into normal human serum was measured, but this time on BAFFR:Fas reporter cells that are more sensitive to BAFF and, unlike BCMA:Fas reporter cells, cannot respond to APRIL. The activity of BAFF 60-mer was decreased by up to two orders of magnitude when it was spiked into normal human serum compared to 60-mer spiked into fetal calf serum (Figure 2A). This could have been due to the presence of shed soluble BAFFR, TACI, and/or BCMA, all of which have been described (Hoffmann et al., 2015; Laurent et al., 2015; Smulski et al., 2017), but pre-depletion of serum on beads coupled to recombinant Fc-BAFF, which could remove soluble TACI, BAFFR, and BCMA (Supplementary Figure 1), did not alter the inhibitory activity (Figure 2A). After serum concentration using an ultrafiltration device with 30 kDa cut off, and exchange of the serum matrix for PBS, all 60-mer inhibitory activity was recovered and enriched in the retained fraction, and none passed into the low molecular weight fraction (Figure 2B). In line with these results, the inhibitory activity recovered after size-exclusion chromatography was in the high-molecular weight fractions, and not in smaller molecular weight Ig- or albumin-containing fractions (Figures 2C,D). It was abolished by heating at 95°C (Figure 2E), but resisted heating at 56°C (Supplementary Figure 2A) and was unaffected by a cocktail of protease inhibitors (Supplementary Figure 2B). The inhibitory activity was consistently found in adult human sera and plasma (Supplementary Figure 2C), and in sera of adult cows (Figure 2F). It was present in varying amounts in sera obtained from human cord blood, but usually lower than in adult sera (Figure 2G). It was particularly low in two cord blood samples of pre-term babies born at gestational weeks 28 or 29 (Figure 2H). It was not present in fetal calf serum and adult mouse sera (Figure 2I).

### A BAFF ELISA Recognizes BAFF 60-mer Only When BAFF Is Captured at pH 5.5

**FIGURE 3:**
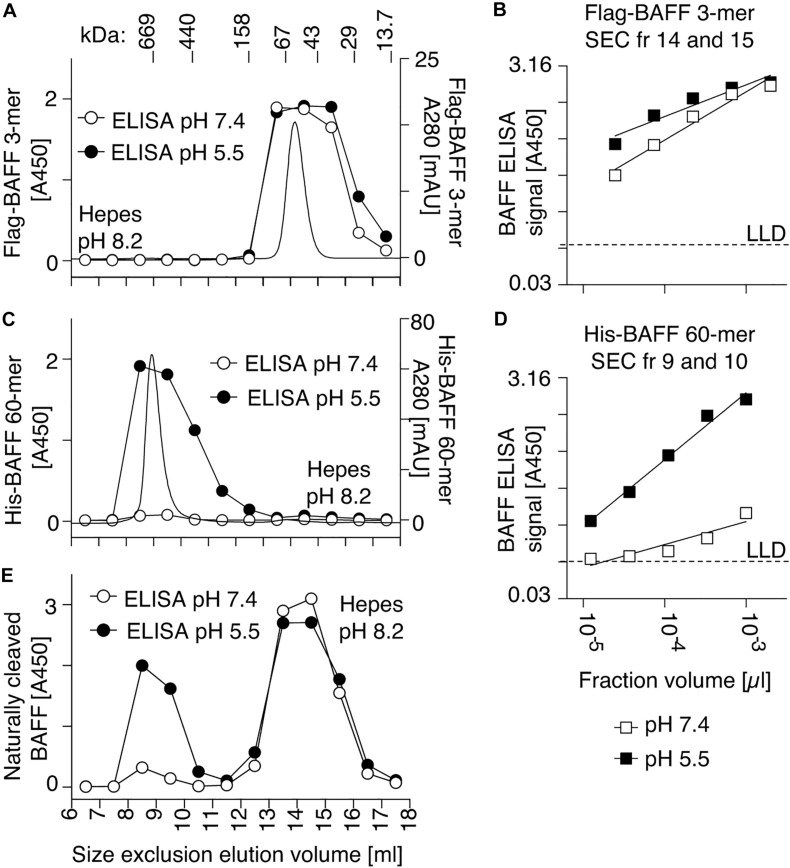
A human BAFF ELISA detects BAFF 60-mer at pH 5.5 but not at pH 7.4. **(A)** 50 μg of Flag BAFF 3-mer was fractionated by SEC at pH 8.2 and detected by on-line UV monitoring (thin line). Fractions were tested by BAFF ELISA with the capture step performed at pH 7.4 (white circles) or pH 5.5 (black circles). **(B)** Titration of Flag-BAFF 3-mer from SEC fractions 14 + 15 measured by BAFF ELISA with capture at pH 7.4 (white squares) or pH 5.5 (black squares). LLD: lowest limit of detection. **(C,D)** Same as panels **(A,B)**, but with 100 μg of His-BAFF 60-mer and His-BAFF 60-mer from SEC fractions 9 + 10. Experiments of panels **(A–D)** were performed once in this format, but pH sensitive detection of BAFF 60-mer was confirmed in 3 more experiments in different formats. **(E)** Same as panels **(A)**, but with naturally cleaved BAFF in concentrated supernatants of 293T cells transfected with full length human BAFF, and with 10 μg/ml BSA in buffer. The experiment was performed twice.

Purified recombinant Flag-BAFF was eluted by size-exclusion chromatography at a size of 67 kDa, slightly higher than its theoretical size of 56 kDa (3.6-mer). It was recognized in the BAFF ELISA at both pH 7.4 and pH 5.5 (Figures 3A,B). In contrast, His-BAFF 60-mer, and the 60-mer fraction of naturally processed full-length BAFF in supernatants of transfected 293T cells, were not recognized at pH 7.4 and only detected at pH 5.5 (Figures 3C–E). This probably indicates that a concealed epitope in BAFF 60-mer becomes available for capture upon acid-dissociation. Thus, the capture of BAFF at pH 5.5 is mandatory to detect BAFF 60-mer.

### The BAFF 60-mer Inhibitory Activity of Human Serum Dissociates BAFF 60-mer Into 3-mer and Is Saturable

**FIGURE 4:**
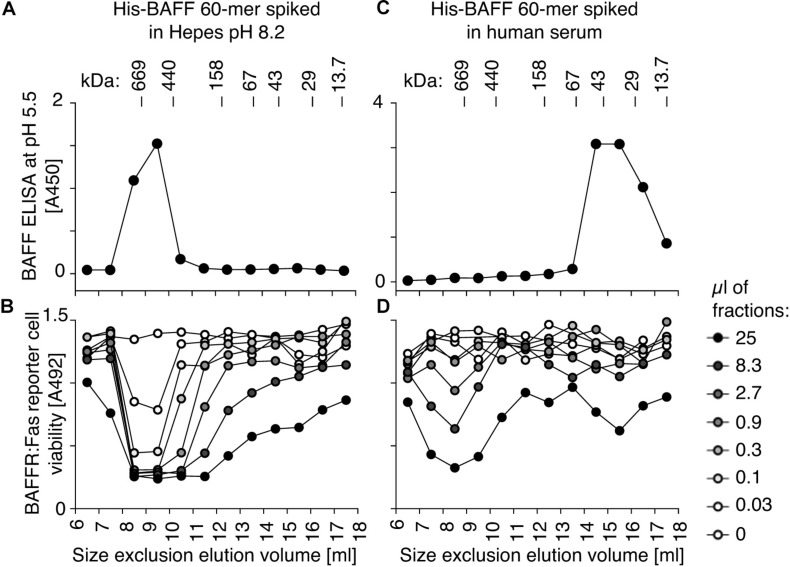
Human serum dissociates His-BAFF 60-mer into less active 3-mer. **(A)** 40 ng of His-BAFF 60-mer in Hepes pH 8.2, 50 μg/ml BSA was size fractionated by SEC and the presence of BAFF in 70 μl of fractions was analyzed by BAFF ELISA with capture at pH 5.5. **(B)** The indicated volumes of the same factions as in panel **(A)** were analyzed for their activity on BAFFR:Fas reporter cells. **(C,D)** Same as panels **(A,B)**, except that the same amount of BAFF 60-mer was spiked into 400 μl of normal human serum at pH ∼8 prior to fractionation by SEC at pH 8.2. The experiments of panels **(A,C)** were performed 3 times, and those of panels **(B,D)** twice.

**FIGURE 5:**
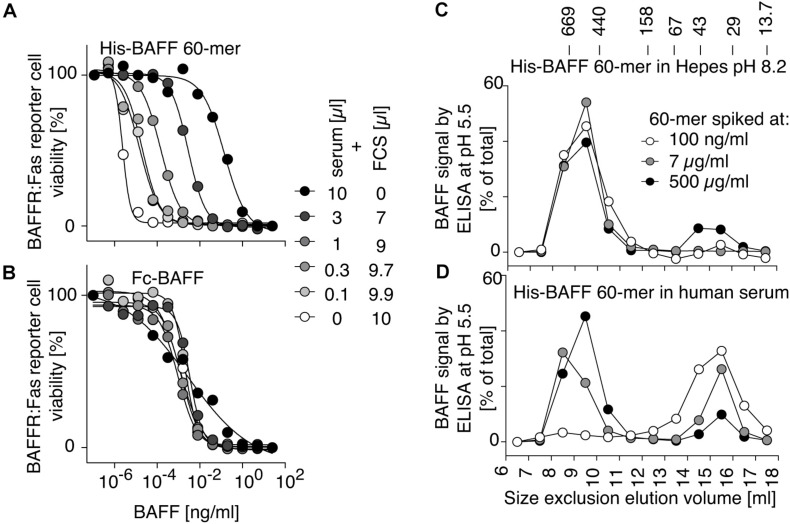
BAFF 60-mer dissociation activity of human serum is saturable. **(A)** The inhibitory activity of normal human serum mixed with FCS at the indicated ratio on His-BAFF 60-mer was tested on BAFFR:Fas reporter cells. **(B)** Same as panel **(A)**, but using Fc-BAFF instead of His-BAFF 60-mer. The experiments of panels **(A,B)** were performed three times. **(C)** His-BAFF 60-mer spiked at 0.1 (white circles), 7 (gray circles), or 500 μg/ml (black circles) in 400 μl of Hepes buffer at pH 8.2 was size-fractionated by SEC at pH 8.2 and analyzed in adequately diluted fractions by BAFF ELISA with capture at pH 5.5. Data is normalized to the total signal in fractions 7 to 18 for each individual run. **(D)** Same as panel **(C)**, except that His-BAFF 60-mer was spiked into 400 μl of normal human serum. The experiment of panels **(C,D)** was performed twice.

BAFF 60-mer spiked into Hepes buffer at pH 8.2 or in human serum was size-fractionated by size-exclusion chromatography and detected in fractions by ELISA at pH 5.5 and by its activity on BAFFR:Fas reporter cells. In Hepes buffer, both protein and activity eluted in high molecular weight fractions, as expected for BAFF 60-mer (Figures 4A,B), but when spiked into serum, BAFF 60-mer protein was recovered at the size of BAFF 3-mer, while the leftover activity was still mainly 60-mer and partially 3-mer, suggesting that highly active 60-mer was almost entirely dissociated to less active 3-mer by exposure to human serum (Figures 4C,D). If serum inhibits BAFF 60-mer by dissociation, then non-dissociable BAFF oligomers such as hexameric Fc-BAFF should be unaffected by serum. Indeed, human serum inhibited BAFF 60-mer in a concentration-dependent manner (Figure 5A), but did not affect the activity of Fc-BAFF (Figure 5B). To demonstrate whether the BAFF 60-mer inhibitory activity was saturable, increasing concentrations of BAFF 60-mer were spiked into a fixed volume of human serum, and then size fractionated. BAFF was then detected by ELISA at pH 5.5 in adequately diluted fractions, and the percentage of total BAFF in each fraction was calculated. BAFF 60-mer spiked into buffer at pH 8.2 eluted as 60-mer (Figure 5C). When 60-mer was spiked at 100 ng/ml in human serum, almost all of it dissociated to BAFF 3-mer. At 7 μg/ml, only about half dissociated into 3-mer, whereas at 500 μg/ml, almost all of it remained 60-mer (Figure 5D). We take these results as a strong indication that although the BAFF 60-mer-dissociating activity in human serum is limited and saturable, it is very high (EC_50_ of about 7 μg/ml) compared to usual circulating BAFF levels.

### Recombinant BAFF 60-mer Activity Resists Affinity Purification but Is Irreversibly Attenuated in Normal Human Serum

To test whether attenuation of BAFF 60-mer activity in human serum is a reversible process, the activity of BAFF 60-mer spiked into different matrices was analyzed before and after affinity purification procedures on immobilized TACI-Fc (atacicept) or belimumab. BAFF 60-mer bound efficiently to atacicept but not to belimumab. About 10% of atacicept-bound BAFF 60-mer activity was recovered after acid elution, neutralization, and buffer exchange to Hepes pH 8.2 (Supplementary Figure 3). However, when BAFF 60-mer was spiked into human serum, very little activity was recovered after affinity purification on atacicept and buffer exchange to Hepes pH 8.2, suggesting that serum inhibition of BAFF 60-mer is irreversible and cannot be reversed by removing serum and reverting back to 60-mer-friendly conditions (Supplementary Figure 3).

### Human Cerebrospinal Fluid Contains BAFF but no BAFF 60-mer Inhibitory Activity

**FIGURE 6:**
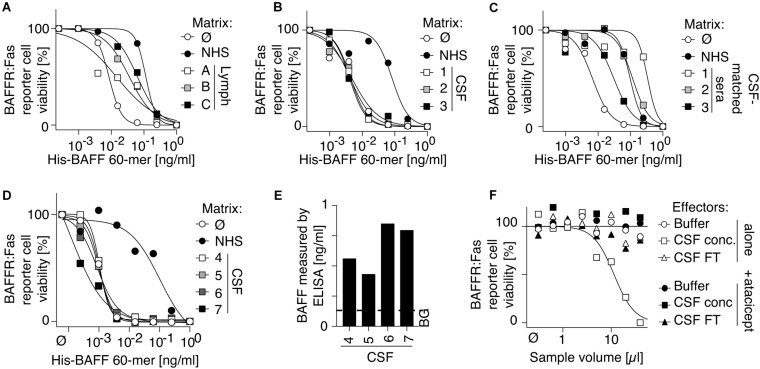
Human cerebrospinal fluid contains BAFF protein and activity, but no BAFF 60-mer inhibitory activity. **(A–D)** Three human samples of lymph exudate **(A)**, 3 human CSF samples of multiple sclerosis patients **(B)**, 3 human sera of patients corresponding to CSF samples of panel **(B,C)** and 4 additional CSF samples from multiple sclerosis patients **(D)** were tested for their inhibitory activity on His-BAFF 60-mer using BAFFR:Fas reporter cells. Experiments in panels **(A–C)** were performed 3 times and that of panel **(D)** once. **(E)** BAFF levels measured by BAFF ELISA with capture at pH 7.4 in the 4 CSF samples of panel **(D)**. This experiment was performed once in this format. Detection of BAFF by ELISA in CSF sample was performed 4 times in different formats in these or other CSF samples. **(F)** A pool of CSF samples from panels **(D,E)** was concentrated 8-fold by ultrafiltration with cut off at 30 kDa. BAFF activity of concentrated CSF (CSF conc), of the filtered fraction of CSF (CSF FT) and of buffer was monitored on BAFFR:Fas reporter cells in the presence or absence of atacicept at 100 ng/ml. The experiment was performed once in this format. Detection of BAFF activity in these or other CSF samples was performed five times in different formats (including in Figure 7).

Owing to its inhibitory activity, human serum might not be the right place to detect BAFF 60-mer. Human lymph exudate also inhibited BAFF 60-mer activity (Figure 6A), although we cannot exclude that this could be due to contaminating serum. In contrast, CSF of three patients with MS did not inhibit BAFF 60-mer activity, while their corresponding sera did (Figures 6B,C). The absence of BAFF-inhibitory activity was confirmed in four more CSF samples (Figure 6D) that all contained low but detectable levels of endogenous BAFF (Figure 6E). After concentration of pooled CSF samples, an atacicept inhibitable BAFF activity was indeed detectable using BAFFR:Fas reporter cells (Figure 6F), raising the possibility that BAFF 60-mer may exist in CSF.

### BAFF in Human Cerebrospinal Fluid Forms 3-mer

**FIGURE 7:**
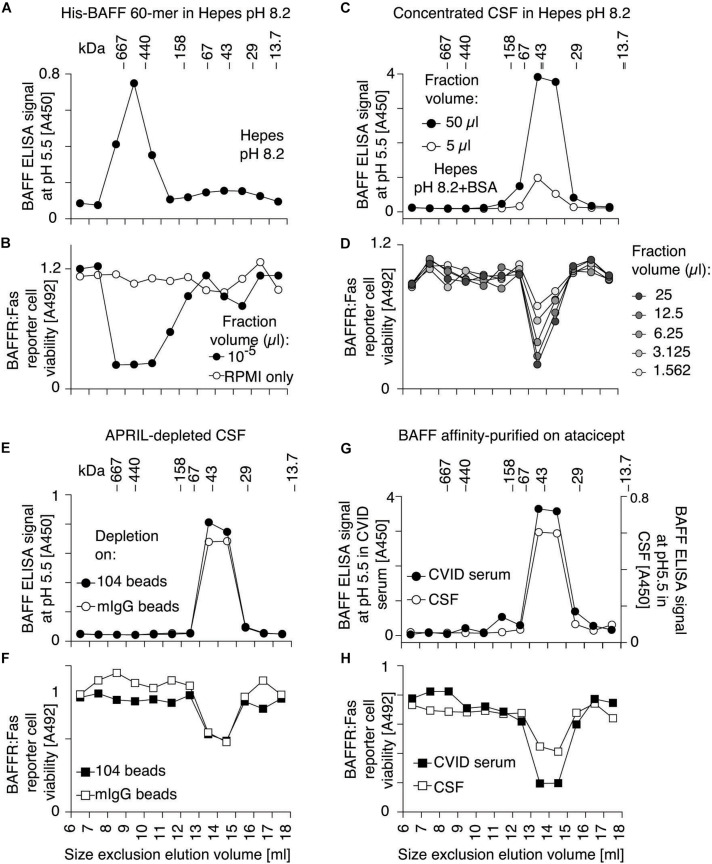
BAFF protein and activity in human CSF has the size of a 3-mer, even after depletion of BAFF-APRIL heteromers or after affinity-purification. **(A)** His-BAFF 60-mer in Hepes buffer at pH 8.2 was fractionated by SEC at pH 8.2 in a buffer with 140 mM NaCl and no BSA. Small aliquots diluted in the same buffer with 30 mM NaCl were lyophilized, dissolved in a tenth of the volume and measured by BAFF ELISA with capture at pH 5.5. The experiment was performed once in this format, and three times in different formats. **(B)** Fractions of panel **(A)** were analyzed for BAFF activity on BAFFR:Fas reporter cells. This experiment was performed twice. **(C)** Same as panel **(A)**, except that 200 μl of an 8-fold concentrated pool of CSF from patients with multiple sclerosis (4–7 in Figures 6D,E) was analyzed instead of His-BAFF 60-mer, and that Hepes buffer contained 30 mM NaCl only, and that fractions were lyophilized and dissolved in a fifth of the initial volume prior to analysis. **(D)** Fractions of panel **(C)** were analyzed for BAFF activity on BAFFR:Fas reporter cells. Experiments of panels **(C,D)** were performed once in this format, and three times in different formats (panels **E–H**). **(E)** CSF depleted on mAb 104, which removes APRIL and BAFF-APRIL heteromers, was size-fractionated at pH 8.2 in the presence of Hepes buffer containing 30 mM NaCl and 10 μg/ml BSA. After lyophilization of fractions and suspension in a tenth of the original volume, BAFF was detected by ELISA with capture at pH 5.5 (black circles). Mock-depleted CSF was also analyzed (white circles). **(F)** Fractions of panel **(E)** were analyzed for BAFF activity on BAFFR:Fas reporter cells. **(G)** BAFF affinity-purified on atacicept from patients with multiple sclerosis (4–7 in Figures 6D,E; white circles), or serum from a CVID patient (black circles) were size fractionated by SEC at pH 8.2. BAFF in fractions was detected by ELISA with capture at pH 5.5. Note that the *Y*-axis scale is different for both samples. **(H)** Fractions of panel **(G)** were tested for BAFF activity on BAFFR:Fas reporter cells. A 4-fold higher fraction volume was used to measure BAFF activity in CSF compared to CVID. Experiments of panels **(E,G)** were performed once. Measures in panel **(F)** were performed twice, from the same fractions. Measures in panels **(F,H)** were performed at 3 different dilutions, one of which is shown.

Pooled CSF samples were concentrated, fractionated by size exclusion chromatography at pH 8.2 and assayed for BAFF content by ELISA at pH 5.5 and on BAFFR:Fas reporter cells. Both assays exclusively detected BAFF at the size of a 3-mer, while a positive control of BAFF 60-mer analyzed under the same conditions eluted with the expected high molecular weight (Figures 7A–D). We hypothesized that a portion of BAFF in CSF could be engaged into BAFF-APRIL heteromers that would inhibit 60-mer formation, but after passage of CSF on an immobilized anti-APRIL antibody able to deplete BAFF-APRIL heteromers, BAFF was still present as 3-mer in CSF (Figures 7E,F). When endogenous BAFF present in CSF or in a BAFF-high serum sample (from a patient with common variable immunodeficiency) was affinity-purified on atacicept prior to size-fractionation at pH 8.2, only BAFF 3-mer was detected, indicating the BAFF in CSF and in CVID serum is not only 3-mer, but also unable to associate as 60-mer under favorable conditions (Figures 7G,H).

### A High Molecular Weight Form of BAFF in Cord Blood

**FIGURE 8:**
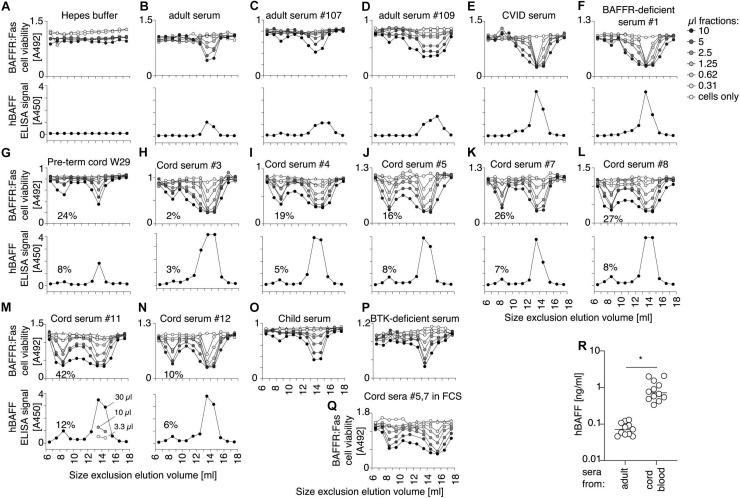
Consistent detection of a high molecular weight form of BAFF in cord blood. Human sera were fractionated by SEC in Hepes pH 8.2, 30 mM NaCl, 10 μg/ml BSA. Fractions were lyophilized, redissolved in one tenth of the volume, and used to monitor BAFF activity on BAFFR:Fas reporter cells in the presence of the activating anti-BAFF antibody 2.81. Fractions were also measured for BAFF content by ELISA with a capture step at pH 5.5. **(A)** Negative control with buffer only. **(B–D)** Normal human adult sera. **(E)** Serum from a CVID patient. **(F)** Serum from a BAFFR-deficient individual. **(G)** Serum from cord blood of a pre-term baby at gestational week 29. **(H–N)** Cord blood sera. Note that serum in panel **(H)** was analyzed before in-depth cleaning of the chromatography system and may contain traces of TACI-Fc. Peak 3-mer fractions in panel **(M)** were measured at 3 different dilutions, showing a linear relationship between signal and dilution. **(O)** Serum from a 7 year-old child. **(P)** Serum from a BTK-deficient patient. **(Q)** Pooled cord blood sera #5 and #7 size fractionated in a column equilibrated in 25% fetal calf serum instead of Hepes buffer pH8.2 **(R)** Concentrations of BAFF were measured by ELISA at pH 7.4 in 10 μl of normal human sera (*n* = 11) and in 3 μl of sera of cord blood (*n* = 12), using Flag-BAFF as a standard. Groups were compared by *t*-test with Welch’s correction. *p < 0.05.

Because fetal calf serum contains less BAFF 60-mer dissociating activity than adult cow serum, we tested whether human cord blood that also contains low dissociating activity may contain BAFF 60-mer. BAFF in fractions of the size exclusion chromatography was monitored by activity using BAFFR:Fas reporter cells, and by ELISA. As BAFFR:Fas reporter cells are highly sensitive to BAFF oligomers (Vigolo et al., 2018), but less so to BAFF 3-mer, they cannot detect low levels of endogenous BAFF 3-mer. Activity assays were therefore systematically performed in the presence of the cross-linking anti-human BAFF mAb 2.81, that we found was able to enhance the activity of BAFF 3-mer (see later). Also, the BAFF ELISA was systematically performed at pH 5.5 in order to detect both 3-mers and 60-mers. This also allows to compare total BAFF protein to activity. Finally, because the chromatography system was also used by our laboratory to purify recombinant BAFF 60-mer or TACI-Fc, the entire system was thoroughly cleaned until no trace of BAFF activity, or BAFF inhibitory activity was detected (Figure 8A). BAFF in normal adult sera was detected as 3-mer, with only traces of higher molecular weight BAFF, and we confirmed that this was also the case for CVID and BAFFR-deficient sera (Figures 8B–F). However, all cord blood sera, including one of a pre-term child at gestational week 29 contained fair proportions of high molecular weight BAFF in addition to 3-mers: up to 13% by ELISA and up to 40% in the activity test (Figures 8G–N). A single child serum was analyzed. It resembled adult serum more than cord serum (Figure 8O). An adult serum from a patient without B cells (BTK deficiency) contained 3-mer only, suggesting that differences observed for high molecular weight BAFF between cord blood and adults was not B cell-related (Figure 8P). In cord sera, despite the presence of the activating antibody, high molecular weight BAFF consistently displayed a higher specific activity compared to BAFF 3-mer, which is one of the characteristics of BAFF 60-mer. We excluded that formation of high molecular weight BAFF would be induced only in Hepes pH8.2, because it was also observed when the column was equilibrated in 25% fetal calf serum instead of Hepes buffer pH 8.2 (Figure 8Q). A direct measure in twelve cord blood samples revealed BAFF levels that were on average13-fold higher than in healthy adult sera (Figure 8R).

### High Molecular Weight BAFF in Cord Blood Can Dissociate Into 3-mers

**FIGURE 9:**
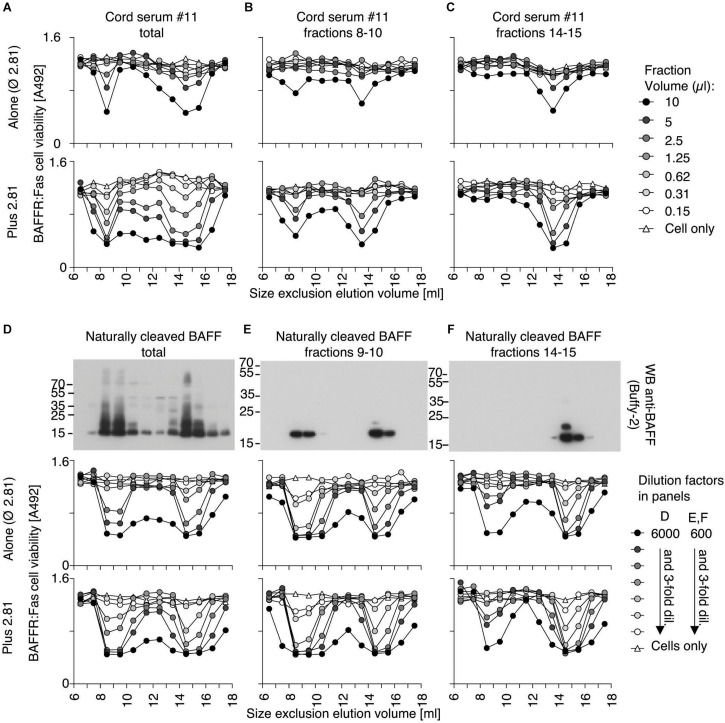
High molecular weight cord blood BAFF and naturally cleaved BAFF 60-mer similarly dissociate into 3-mer. **(A)** Cord serum was analyzed as described in Figure 8, but in the absence (top panel) or presence (bottom panel) of activating anti-BAFF 2.81 antibody. **(B)** Fractions 8–10 of panel **(A)** were concentrated and re-fractionated by SEC. Lyophilized fractions were analyzed on BAFFR:Fas reporter cells without or with activating anti-BAFF 2.81 antibody. **(C)** Same as panel **(B)**, but with fractions 14–17 from panel **(A)**. **(D)** Conditioned supernatants of 293T cells transiently transfected with full-length human BAFF were size-fractionated by SEC in PBS pH 7.4. Fractions were analyzed by anti-BAFF (Buffy-2) western blot (top panel), or by activity on BAFFR:Fas reporter cells without (middle panel) or with 2.81 (bottom panel). **(E)** Fractions 9–10 of panel **(D)** were concentrated by affinity-purification on atacicept and analyzed again by SEC in Hepes at pH 8.2, Western blot and activity tests. **(F)** Same as panel **(E)**, but with affinity-purified fractions 14–17 of panel **(D)**. Note that affinity-purification steps render results of panels **(E,F)** not directly comparable with those of panels **(B,C)**.

Size exclusion chromatography fractions of one of the cord blood samples were monitored for BAFF activity with or without activating antibody. As expected, the activity of BAFF 3-mer was enhanced with the activating antibody, but high molecular weight BAFF was activated too (Figure 9A), suggesting it might have dissociated into 3-mers. When the high molecular weight BAFF fraction was fractionated again, about 60% had dissociated into 3-mers while the remaining was still big (Figure 9B). On the contrary, the trimeric fraction did not detectably re-associate into multimers (Figure 9C). Naturally processed full-length recombinant BAFF yielded BAFF 3-mer and 60-mer in roughly similar quantities, as detected by Western blot (Figure 9D). Unexpectedly, the activity of the 60-mer on reporter cells was similar to that of BAFF 3-mer in terms of signal and of response to ligand (Figure 9D), which could be attributed at least in part to an equilibrium between 60-mer and 3-mer after size separation. Re-fractionation of BAFF 60-mer indeed yielded again 3-mer and 60-mer in equivalent amounts (Figure 9E), while the 3-mer remained essentially 3-mer, with moderate amounts of 60-mer detected by the activity test, but not by Western blot (Figure 9F). Taken together, these results show that high molecular weight BAFF in cord blood can dissociate into 3-mers similarly to naturally cleaved BAFF 60-mer.

### Epitopes Concealed in Recombinant BAFF 60-mer Are Accessible in High Molecular Weight BAFF From Cord Blood

**FIGURE 10:**
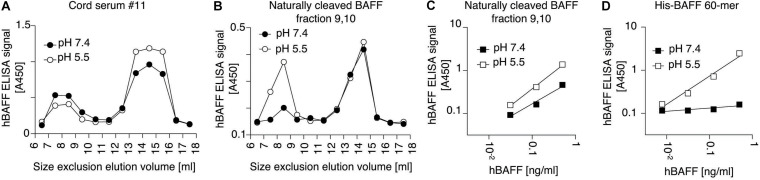
High molecular weight cord blood BAFF is detected by BAFF ELISA also at pH 7.4. **(A)** A cord serum sample was size-fractionated by SEC. Fractions were immediately analyzed by BAFF ELISA with a short capture time (30 min at 4°C instead of 3 h at room temperature). **(B)** Same as panel **(A)**, except that the same cord serum sample was first depleted from endogenous BAFF and spiked with 5 ng/ml of recombinant, naturally cleaved BAFF 60-mer. **(C)** Naturally cleaved recombinant BAFF 60-mer used in panel **(A)** (but not spiked in serum) was measured in the BAFF ELISA with capture steps at pH 5.5 or pH 7.4. **(D)** Same as panel **(C)**, but with recombinant His-BAFF 60-mer.

B cell activating factor 60-mer forms a defined, organized structure, with receptor-binding site always exposed at the surface, while other surfaces are always pointing inside of the 60-mer, or are buried in 3-mer to 3-mer interactions (Liu et al., 2002, 2003). Thus, antibodies against BAFF 3-mers do not necessarily recognize BAFF 60-mer. Belimumab is a well characterized example of an antibody that cannot recognize BAFF 60-mer (Shin et al., 2018; Vigolo et al., 2018). Our results also suggest that the capture antibody of the BAFF ELISA does not recognize BAFF 60-mer at pH 7.4, unless BAFF is first (presumably) dissociated into 3-mer at pH 5.5 (Figures 3C,D). We tested whether high molecular weight BAFF in cord sera would escape recognition by antibodies specific for BAFF 3-mer. Thus, a serum of cord blood was analyzed in parallel with a standard of naturally cleaved BAFF 60-mer added in the same matrix. For this purpose, cord-blood was first depleted from endogenous BAFF with immobilized TACI-Fc, then supplemented with a close-to-endogenous concentration of recombinant 60-mer purified from naturally cleaved BAFF. These samples were size-fractionated by SEC. Fractions were immediately added to ELISA plates at 4°C so that the capture step was completed in less than an hour post-elution. High molecular weight BAFF and BAFF 3-mer in cord blood were detected in the BAFF ELISA at both pH, suggesting it does not contain BAFF 60-mer (Figure 10A). Recombinant 60-mer spiked into the same matrix eluted as 60-mer and 3-mer. As expected, 60-mer was detected at pH 5.5, but poorly at pH 7.4, while the 3-mer was detected at both pH (Figure 10B). This suggests that high molecular weight BAFF in cord blood is different from naturally cleaved, recombinant BAFF 60-mer. Further controls indicated that BAFF 3-mer in purified naturally cleaved BAFF 60-mer was already present before spiking the depleted serum. Indeed, the ELISA recognized this standard at pH 5.5, as expected, but also to a fair extent at pH 7.4, while the more stable His-BAFF 60-mer was recognized at pH 5.5, but not or only weakly at pH 7.4 (Figures 10C,D).

**FIGURE 11:**
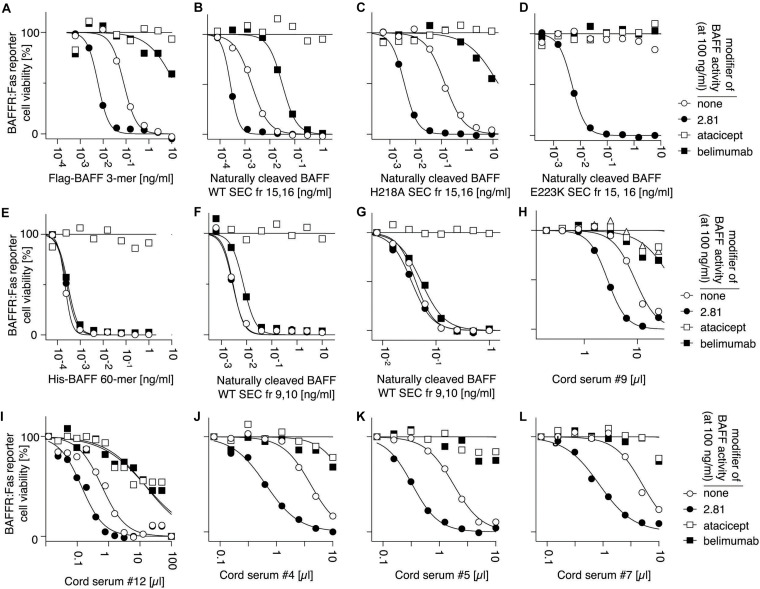
BAFF in cord blood is inhibited by belimumab, as measured in a cell-based assay that distinguishes BAFF 60-mer from BAFF 3-mer. Activities of different forms of recombinant BAFF were monitored on BAFFR:Fas reporter cells in the presence of the following modifiers: (none, white circles), an activating anti-BAFF monoclonal antibody (2.81, black circles), a TACI-Fc decoy receptor (atacicept, white squares) or a blocking anti-BAFF monoclonal antibody (belimumab, black squares). **(A)** Flag-BAFF 3-mer. **(B)** Small molecular weight form (SEC fractions 15 + 16, 3-mer) of naturally processed WT BAFF in supernatants of transfected 293T cells. **(C)** Small molecular weight form (SEC fractions 15 + 16, 3-mer) of naturally processed BAFF with mutation H218A in the flap that prevents 60-mer formation but not signaling through BAFFR. **(D)** Small molecular weight form (SEC fractions 15 + 16, 3-mer) of naturally processed BAFF with mutation E223K in the flap region that prevents formation of 60-mer and signaling through BAFFR. **(E)** His-BAFF 60-mer. **(F)** High molecular weight form (SEC fractions 9 + 10, 60-mer) of naturally processed WT BAFF in supernatants of transfected 293T cells. **(G)** Same as panel **(F)**, but from another experiment. **(H–L)** Cord serum samples. Experiments in panels **(A–E)** and **(F + G)** were performed five, three, two, two, five, and six times, respectively. Experiments in panels **(H–L)** were performed once. Data for panels **(A–F)**, **(G–H)**, **(I)**, and **(J–L)** were collected in independent experiments.

We next tested whether high molecular weight BAFF in cord blood would be resistant to belimumab, as would be expected for BAFF 60-mer, using BAFFR:Fas reporter cells. The development and the characteristics of this assay are described in detail in the Supplementary Material (Supplementary Figures 4–7). Briefly, BAFF-containing samples were titrated on reporter cells, in a medium at pH 8.2 to favor 60-mers, in four different conditions: (i) without modifiers, (ii) with anti-BAFF antibody 2.81 that activates BAFF 3-mer by cross-linking but has no effect on BAFF 60-mer, (iii) with atacicept that inhibits all forms of BAFF, and (iv) with belimumab that inhibits BAFF 3-mer, but minimally affects BAFF 60-mer. This test permits the detection of recombinant His-BAFF 60-mer in the pg/ml range, even in the presence of an excess of Flag-BAFF 3-mer (Supplementary Figure 5). Thus, Flag-BAFF that exclusively forms 3-mer (Schneider et al., 1999), is activated about 10-fold by 2.81, but inhibited by belimumab and atacicept (Figure 11A). Similar results were observed for the 3-mer fraction of naturally cleaved WT BAFF, or of naturally cleaved BAFF with the H218A mutation that prevents 60-mer formation (Vigolo et al., 2018; Figures 11B,C). With the more “severe” mutation E223K that abolishes signaling ability, but not receptor binding (Vigolo et al., 2018), naturally cleaved BAFF was fully dependent on the cross-linking action of 2.81 (Figure 10D). In contrast, recombinant His-BAFF 60-mer was active on its own, was not further activated by 2.81, was fully resistant to inhibition by belimumab, but sensitive to inhibition by atacicept (Figure 11E). Similar results were obtained with the 60-mer fraction of naturally cleaved WT BAFF, except that the activity was overall lower, and that it was weakly inhibited by belimumab, as anticipated if a fair proportion of less active 3-mer would be inhibited in this preparation (Figures 11F,G). Cord blood samples consistently behaved as standards of BAFF 3-mer in this assay, and there was no difference between inhibitions by belimumab or atacicept (Figures 11H–L). Given the proportion of high molecular weight BAFF observed after SEC (Figure 8), if this high molecular weight BAFF would have had the activity of His-BAFF 60-mer, it should have been detected in this assay. We conclude that under conditions of this assay, high molecular weight BAFF in cord blood is recognized and inhibited by belimumab. In only one cord sample did we detect a BAFF activity that was resistant to belimumab and in good agreement with the percentage detected by ELISA post SEC (Supplementary Figure 7). The result could not be repeated because of insufficient amounts of sample. Taken together, these results indicate that the high molecular weight BAFF in cord blood is recognized by two antibodies that cannot bind recombinant BAFF 60-mer.

## Discussion

The ability of BAFF to form 60-mer is a likely evolutionary conserved feature, since the length and critical residues of the flap region are conserved across species (Bossen et al., 2008). Although the presence of mouse BAFF 60-mer in BAFF transgenic and TACI-ko mice (Bossen et al., 2008) and human BAFF 60-mer in conditioned medium of U937 cells (Cachero et al., 2006) were reported, there is still no evidence showing the existence of BAFF 60-mer in human. BAFF 60-mer is different from BAFF 3-mer, not only in terms of size and activity, but also with regards to recognition by different antibodies. We took advantage of some of these differences to develop test systems which are able to discriminate between the activities of BAFF 3-mers and 60-mers. We also adapted an ELISA to enable recognition of both 3-mer and 60-mer, and not only 3-mer as is the case with the standard protocol. A potentially criticisable aspect of the present study is the use of the surrogate Fas signaling pathway in reporter cells, but the sensitivity of this assay is high, with an EC50 of 0.05 pg/ml, or 0.005 pg/100 μl (Supplementary Figure 5A). The molecular mass of BAFF 60-mer being 1,100 kDa, this is equivalent to about 3,000 molecules of 60-mer per well, i.e., less than one 60-mer per reporter cell. Despite the sensitivity of this assay, BAFF 60-mer remained undetected in adult human sera, even in those of a patient with CVID and of a patient with BAFFR-deficiency, in which circulating BAFF levels are up to 500 times higher than in normal human serum. Moreover, we found that serum is not a favorable environment for BAFF 60-mer as it considerably, but not totally, decreases its activity. BAFF 60-mer is known as a pH-sensitive structure which dissociates into less active trimers at acidic pH (Liu et al., 2002; Cachero et al., 2006). However, the 60-mer inhibitory activity was not due to the pH, salt concentration or other physical properties of human serum, as serum after ultrafiltration contained no inhibitory activity. This observation raised the question if BAFF 60-mers might be inhibited by soluble extracellular domains of BAFFR, TACI, and/or BCMA, all of which can be shed from the transmembrane forms of the receptor (Smulski and Eibel, 2018). However, this hypothesis seems to be very unlikely. First, because this inhibitory activity could not be depleted on immobilized Fc-BAFF; second, because the inhibitory activity has a high molecular weight incompatible with that of soluble receptors, third, because it is inconceivable how soluble receptors should specifically target BAFF 60-mer and not Fc-BAFF, and fourth, because atacicept, which is a soluble dimeric form of TACI, binds to BAFF 60-mer without dissociating it (Bossen et al., 2008). We found that the high molecular weight, BAFF 60-mer dissociating activity could not bind to Fc-BAFF, but we do not exclude the possibility that it could bind specifically to BAFF 60-mer, for example if it recognizes the flap-flap interface. When we realized that human serum efficiently dissociated recombinant BAFF 60-mer into 3-mer, we immediately thought that the residual activity was due to the newly formed, less active BAFF 3-mer. This was, however, not the case, as most of this residual activity had the size and properties of BAFF 60-mer (Figure 4D and Supplementary Figure 4B). Whether longer incubations in serum would have destroyed this residual 60-mer activity, or whether there is a fraction of serum-resistant recombinant BAFF 60-mer remains to be investigated.

As BAFF and APRIL can heteromerize (Roschke et al., 2002; Dillon et al., 2010; Schuepbach-Mallepell et al., 2015), and as APRIL is devoid of the flap region that in BAFF is required for 3-mer to 3-mer interactions and 60-mer formation, it is possible that low concentrations of BAFF-APRIL heteromers could prevent 60-mer formation, explaining why all endogenous BAFF in serum is detected as 3-mer. However, depletion of APRIL, homomers and heteromers, with an anti-APRIL antibody did not restore 60-mer formation. This alone is, however, insufficient to discard the hypothesis that BAFF-APRIL heteromers would interfere with 60-mer formation because we find that endogenous BAFF 3-mer and BAFF 3-mer dissociated from recombinant 60-mer cannot re-associate into 60-mer, even after APRIL has been removed. In this context, it still remains to be solved why BAFF 3-mers originating from dissociated 60-mers and why endogenous human BAFF cannot assemble into 60-mers even in serum- or CSF-free conditions. An appealing hypothesis is that the flap region (or other portions of BAFF that interact with the flap) is modified by proteolytic processing. This would explain specific loss of activity of BAFF 60-mer, but not other forms of BAFF. Disruption of one flap out of 60 is in principle sufficient to prevent 60-mer formation. However, the inhibitory activity was not decreased when serum was first treated with a mix of protease inhibitors (Supplementary Figure 2B). An alternative mechanism could be a conformational change in the flap region, such as the one observed in one of the BAFF monomers in the crystal structure of the APRIL-BAFF-BAFF heteromer (Schuepbach-Mallepell et al., 2015). The flap has a defined structure that is virtually identical in all other available crystal structures, including those where flap-flap interactions are prevented by the Fab fragment of belimumab (Shin et al., 2018; Vigolo et al., 2018). There is no doubt that the marked refolding of the long loop of the “canonical” flap into the beta-hairpin seen in the crystal structure of the heteromer would abrogate 60-mer formation.

To try and answer the main question of this study, namely the detection of BAFF 60-mer in human body fluids, we investigated different samples in search of one unable to dissociate recombinant 60-mer. The implication of BAFF in autoimmune diseases such as MS has been studied for years (Kannel et al., 2015). While the transcript levels of BAFF are clearly elevated in active MS lesions (Krumbholz et al., 2005), data about CSF levels of BAFF in MS are not consistent. Some studies found elevated BAFF (Ragheb et al., 2011; Wang et al., 2012; Quan et al., 2013) in MS, others did not (Krumbholz et al., 2005; Kowarik et al., 2012). The BAFF levels in the CSF are influenced not only by local production, but also by consumption, and soluble receptors. The CSF of MS patients contains a variable number of B cells (Stangel et al., 2013) and it is plausible that the CSF levels of BAFF are also determined by consumption of B cells as are the blood levels of BAFF (Kreuzaler et al., 2012). Further, in the CSF of MS patients, the soluble receptors sBCMA and sTACI are elevated and function as decoys (Hoffmann et al., 2015; Laurent et al., 2015).

Here we show that CSF from patients with MS are devoid of BAFF 60-mer dissociating activity. Despite this, endogenous BAFF in CSF was exclusively present as 3-mer, even after purification on atacicept and size-fractionation at basic pH in CSF-free conditions. About 80% of the proteins in the CSF are derived from blood, 19% from the meninges and only 1% from cells in the brain (Stangel et al., 2013). Since the CSF from patients without inflammation in the brain contains BAFF at a similar level as the CSF from MS patients (Krumbholz et al., 2005; Kowarik et al., 2012), we would assume that the majority of the BAFF in the CSF also in MS patients is derived from blood, an hypothesis that would fit with our observations that the CSF contains BAFF-3mer, and that serum permanently transforms BAFF 60-mer into BAFF 3-mer. In addition to CSF, we find that fetal calf serum do not contain dissociating activity, while adult cow sera does. A partially similar situation was observed in humans, with high levels of dissociating activity in adult plasma or serum, lower levels in the umbilical blood of neonates, and even lower levels in two cord blood samples from pre-term babies. In mice, we found no dissociating activity for BAFF 60-mer in adult serum. The mouse BAFF gene contains an additional 30 amino acids at the N-terminus of the soluble form that likely prevents efficient formation of 60-mer. We hypothesize that a destabilization activity would not be required in mouse serum if its goal is to prevent systemic action of BAFF 60-mer. It was previously determined that administration of BAFF 3-mer into BAFF-ko mice restored B cell populations, but not expression of CD23, while administration of BAFF 60-mer restored both, suggesting that BAFF 60-mer may fulfill specific roles (Bossen et al., 2011).

Previous studies reported BAFF levels that were two-fold higher in cord blood compared to maternal blood, although these levels were not maintained in one- or four-month-old babies, suggesting that BAFF could be produced by the placenta (Bienertova-Vasku et al., 2015; Lundell et al., 2015). Interestingly, BAFF was higher in cord blood of babies whose mothers were exposed to dairy farm environment, correlating with more rapid B cell maturation later in childhood and decreased risk of developing allergies (Lundell et al., 2015). Here we find that (i) cord blood contains high levels of BAFF, greater than 10-fold more than in adults, (ii) cord blood usually contains lower levels of BAFF 60-mer dissociating activity, and (iii) cord blood consistently contains up to 10% of a high molecular weight form of BAFF with some, but not all properties of BAFF 60-mer. In particular, this high molecular weight BAFF had a size very similar to that of BAFF 60-mer, i.e., big but still included into the active range of the size exclusion column. It was more active than the fraction of BAFF 3-mer contained in the same sample and could dissociate into 3-mer. These properties would not be expected from a random protein aggregate. However, our data strongly indicate that this high molecular weight BAFF lacked two important features of recombinant BAFF 60-mer: its pH sensitivity in the BAFF ELISA test, and its resistance to inhibition by belimumab. Interestingly, both of these features rely on the inaccessibility of antibody epitopes in BAFF 60-mer, suggesting that they are already accessible, or become rapidly accessible to antibodies in high molecular weight BAFF of cord blood. We excluded the confounding effect of BAFF 60-mer dissociating activity in serum by experiments of depletion and spiking. In addition, specific depletion of APRIL and heteromers did not decrease levels of high molecular weight BAFF, excluding the hypothesis that it may contain BAFF APRIL heteromers (unpublished observations). If high molecular weight BAFF in cord blood is not comparable to recombinant BAFF 60-mer, then what is its molecular nature? In a first scenario, BAFF 60-mer would never form *in vivo*. High molecular weight BAFF would be a complex of undefined nature, such as BAFF 3-mer bound to auto-antibodies or to any other big-sized partner, which would, however, not prevent BAFF activity. This would raise questions of why non-neutralizing anti-BAFF auto-antibodies should be present in cord blood, and similar hard-to-answer questions. In a second scenario, BAFF 60-mer could be formed *in vivo*, most probably locally after its synthesis by BAFF-producing cells. BAFF 60-mer would not be meant to act systemically, and thus would be dissociated into less active 3-mer. This inactivation may proceed through less stable, easy-to-dissociate BAFF 60-mer intermediates. Perhaps one or just a few flaps would adopt a different conformation (Schuepbach-Mallepell et al., 2015) that would render internal epitopes accessible. Binding of just one antibody, or perhaps even a receptor, would quickly dissociate the complex. Two different forms of recombinant BAFF 60-mer, one made in bacteria (His-BAFF 60-mer) and one made from naturally cleaved full-length BAFF expressed in 293T cells, seem to have different stabilities as judged by the proportion of 3-mer released from these structures at pH 8.2 (e.g., Figures 3C, 4A, vs. Figures 3E, 9D–F). Thus, formation of even less stable forms might be considered. Our data so far do not allow distinguishing between these two models, and in view of the minute amounts of BAFF available in these samples, it might be technically challenging to do so. Perhaps more information about a putative function of BAFF 60-mer *in vivo* could come from genetic models in which BAFF 60-mer can or cannot form. Our data, however, demonstrate that clinical BAFF inhibitors will neutralize BAFF in the circulation: highly active forms of BAFF 60-mer are unlikely to be predominant in blood or in CSF, and even the high molecular weight form of BAFF detected in cord blood can be inhibited by both belimumab and atacicept.

In summary, with the help of sensitive tools developed for the characterization of BAFF 60-mer in biologic fluids, we demonstrated the exclusive presence of BAFF 3-mer in adult human serum and CSF samples, and detected a high molecular weight form of BAFF with some but not all properties of BAFF 60-mer in cord blood. In addition, an activity that dissociates BAFF 60-mer into trimers was identified, which is higher in adult serum than in cord blood. Advancing knowledge on the endogenous forms of BAFF is relevant in view of its elevated levels in various disorders (Cheema et al., 2001; Zhang et al., 2001; McCarthy et al., 2013; Xin et al., 2013; Li et al., 2014; Kannel et al., 2015; Salazar-Camarena et al., 2016; Steri et al., 2017) and the use of BAFF antagonists with different ligand specificities in the clinic or in clinical trials.

## Data Availability Statement

The raw data supporting the conclusions of this article will be made available by the authors, without undue reservation, and are available as a data set doi: 10.5281/zenodo.4141692.

## Ethics Statement

The studies involving human participants were reviewed and approved by Ethics Committee of the Medical University of Vienna, Austria (EK Nr: 1845/2015). Human systemic lupus erythematosus serum samples were from patients who were enrolled in the randomized, double-blind, APRIL-SLE trial, but before they received any treatment with atacicept (ClinicalTrials.gov Identifier NCT00624338). Cerebrospinal fluid (CSF) samples from MS patients were provided by the Institute of Clinical Neuroimmunology, Munich. This was approved by the Ethical Committee of the Medical Faculty of Ludwig-Maximilians-Universität München. Work with mice was performed according to Swiss Federal Veterinary Office guidelines, and under the authorization of the Office Vétérinaire Cantonal du Canton de Vaud (authorization 1370.7 to PS). Cow sera: this specific study had been approved by the IACUC of Lower Saxony, the state veterinary office Niedersächsisches Landesamt für Verbraucherschutz und Lebensmittelsicherheit, Oldenburg, Germany (registration number 33.42502-05-04A247).

## Author Contributions

PS and ME designed experiments. ME, LW, and PS performed experiments. ME and PS wrote the manuscript. EM, HE, ODo, ODi, HS, DS, DT, ÖY, and ES provided essential reagents. All authors reviewed the results and approved the final version of the manuscript.

## Conflict of Interest

PS receives research funds from Merck KGaA for related research, and has a licence agreement with Adipogen Life Sciences. ODo is employee of Adipogen Life Sciences. ÖY is employee of Merck KGaA. ES was employee of EMD Serono. The remaining authors declare that the research was conducted in the absence of any commercial or financial relationships that could be construed as a potential conflict of interest.

## Abbreviations

BAFF: B cell activating factor of the TNF family
APRIL: A proliferation-inducing ligand
BAFFR: BAFF receptor
TACI: Transmembrane activator and CAML interactor
BCMA: B cell maturation antigen
SLE: systemic lupus erythematosus
CVID: common variable immunodeficiency
MS: multiple sclerosis
SEC: size exclusion chromatography
CSF: cerebrospinal fluid
FCS: fetal calf serum
BSA: bovine serum albumin.
