# Supplementary material for: BAFF 60-mer, and Differential BAFF 60-mer Dissociating Activities in Human Serum, Cord Blood and Cerebrospinal Fluid
Source: Front Cell Dev Biol. 2020 Nov 6;8:577662. doi: 10.3389/fcell.2020.577662 (PMC7677505; doi:10.3389/fcell.2020.577662)
Supplement: Supplementary file 1 [file Data_Sheet_1.pdf]

SUPPLEMENTARY INFORMATION

BAFF 60-mer, and differential BAFF 60-mer dissociating activities in human serum, cord blood and cerebrospinal fluid.

Mahya Eslami, Edgar Meinl, Hermann Eibel, Laure Willen, Olivier Donzé, Ottmar Distl, Holm Schneider, Daniel E. Speiser, Dimitrios Tsiantoulas, Özkan Yalkinoglu, Eileen Samy and Pascal Schneider

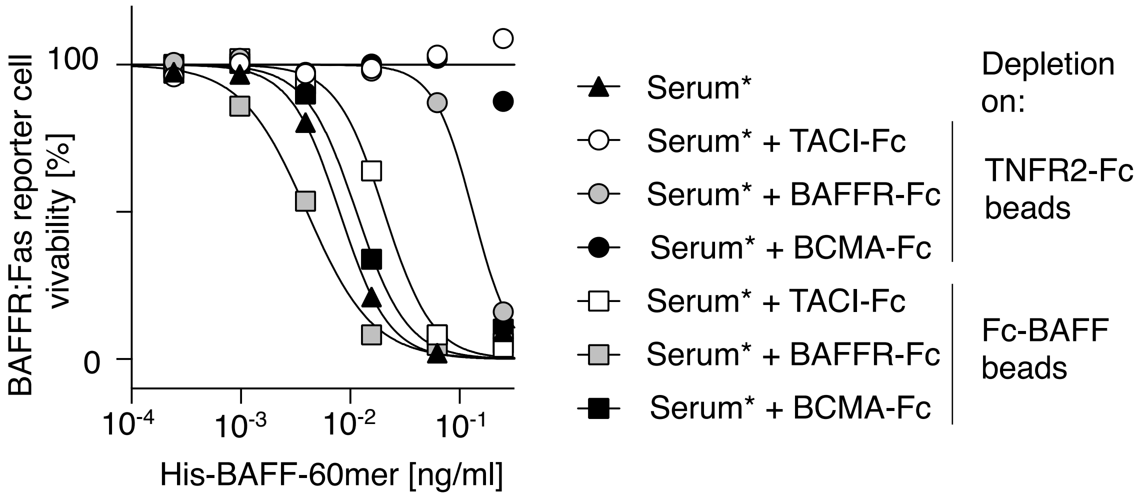

Supplementary Figure 1 (related to Fig. 2A). **Immobilized Fc-BAFF can deplete soluble BAFFR, TACI and BCMA spiked into normal human serum.**
Normal human serum was first depleted on immobilized TACI-Fc to remove endogenous BAFF and APRIL (serum*). Serum* was then left alone or supplemented with soluble TACI-Fc, BAFFR-Fc or BCMA-Fc. Spiked sera were depleted either with immobilized Fc-BAFF, or immobilized TNFR2-Fc as a negative control. The amount of active receptors-Fc remaining in serum post-depletiion was measured by their ability to inhibit recombinant BAFF. For this purpose, recombinant BAFF (His-BAFF-60-mer) was added to BAFFR:Fas reporter cells at the indicated concentrations, in the presence of serum* alone, or serum* spiked with receptors-Fc and depleted on TNFR2-Fc or Fc-BAFF. This experiment was performed once.

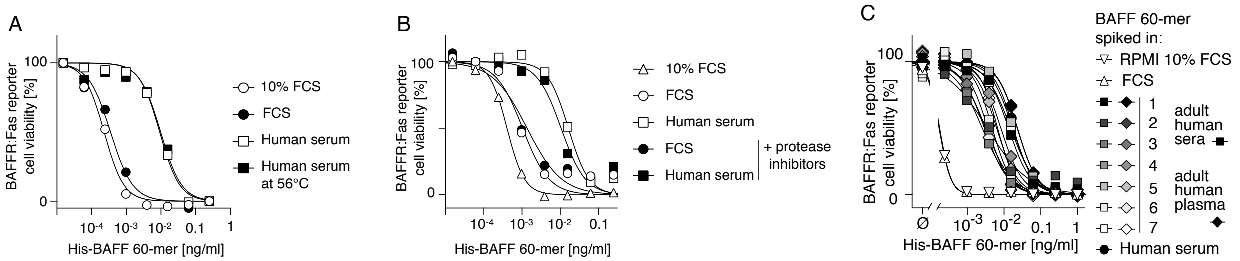

Supplementary figure 2 (related to Fig. 2). **BAFF 60-mer inhibitory activity resists protease inhibitors, heating at 56°C and is present in serum and plasma.**

(A) His-BAFF 60-mer was titrated on BAFFR:Fas reporter cells in medium alone (10% FCS) or in the presence of human serum ± decomplementation for 30 min at 56°C. **(B)** His-BAFF 60-mer was titrated on BAFFR:Fas reporter cells in medium alone (10% FCS) or supplemented with FCS ± protease inhibitors, or supplemented with human serum ± protease inhibitors. **(C)** Seven sera and matched plasma from healthy donors were analysed with the indicated controls. Experiments A was performed twice. Experiment B were performed once. Experiment C was performed 7 times for adult sera and once for plasma.

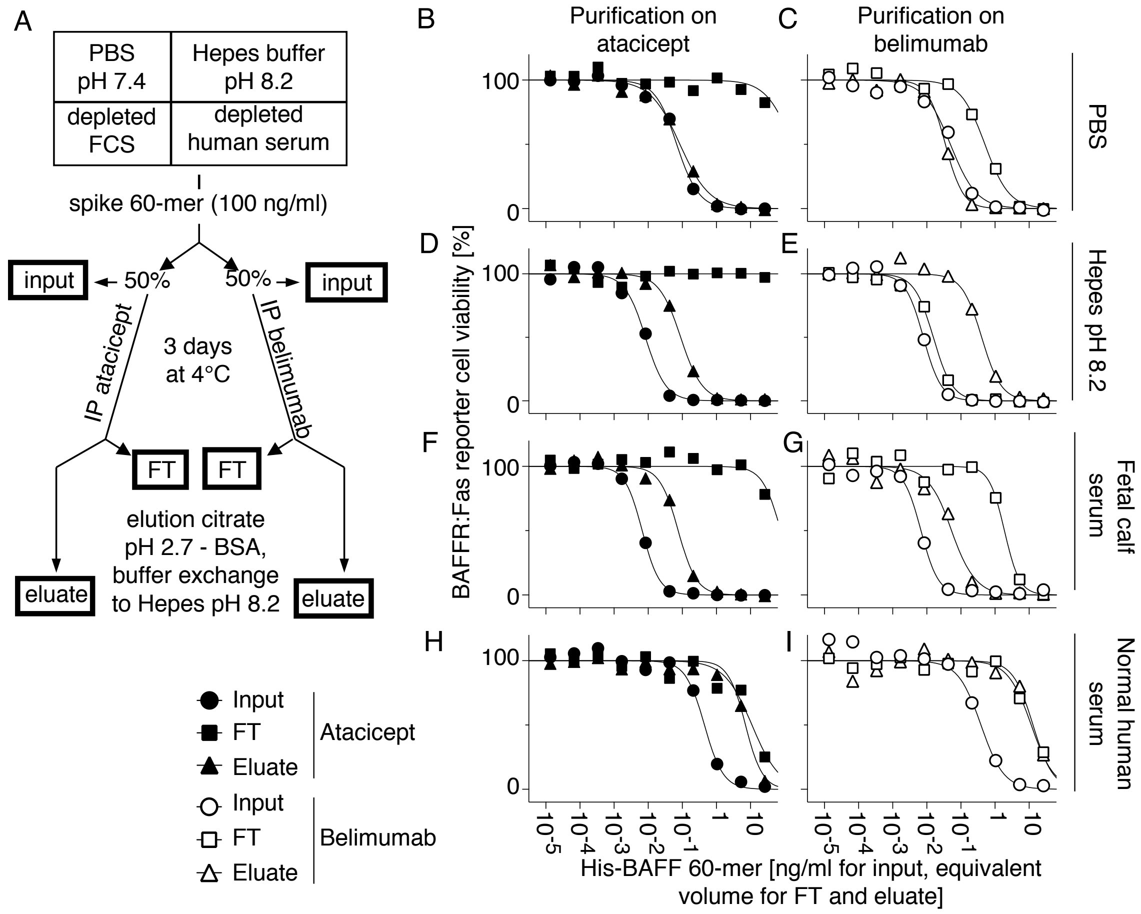

Supplementary figure 3. **Recovery of BAFF 60-mer activity spiked into different matrices by affinity purification on atacicept or belimumab.**

(A) Scheme of the experiment showing the four matrices (PBS pH 7.4, Hepes pH 8.2, FCS pre-depleted on atacicept and NHS pre-depleted on atacicept) in which His-BAFF 60-mer was spiked at 100 ng/ml. The main steps of the affinity purifications on immobilized atacicept or belimumab are also shown. FT: flow through. **(B-I)** BAFF spiked into PBS **(B-C)**, Hepes buffer pH 8.2 **(D-E)**, FCS **(F-G)** or NHS **(H-I)** was affinity purified on immobilized atacicept **(B, D, F, I**, black symbols**)** or immobilized belimumab **(C, E, G, I,** white symbols**)**, and the three fractions of each affinity purification, namely input (circles), flow through (FT, squares) and eluate (triangles) were quantified for BAFF activity by titration on BAFFR:Fas reporter cells. This entire experiment was performed twice.

Recombinant BAFF 60-mer activity resists affinity purification but is irreversibly attenuated in normal human serum.

To test whether attenuation of BAFF 60-mer activity in human serum is a reversible process, the activity of BAFF 60-mer spiked into different matrices was analysed before and after affinity purification procedures. BAFF 60-mer was slightly more active in Hepes buffer pH 8.2 than in PBS pH 7.4, which can reflect more extensive 60-mer formation at pH 8.2 (Fig. S3A-E, black and white circles). As expected, BAFF 60-mer remained active in foetal calf serum (FCS), but was

markedly less so in the presence of human serum (Fig. S3F-I, black and white circles). When BAFF in PBS, in Hepes pH 8.2 or in FCS was affinity-purified on immobilized TACI-Fc (atacicept), activity was removed from the flow through of the purification, indicating that immobilized atacicept captured all BAFF (Fig. S3B,D,F,H, black squares). After acid elution and buffer exchange to pH8.2, about 10% of the initial activity was recovered (Fig. S3B, D, F, H, black triangles). The apparent total recovery of activity for BAFF 60-mer in PBS is probably due to the more 60-mer-favourable conditions post-purification (pH 8.2) compared to input (pH 7.4) in this particular case (Fig. S3B, black circles and triangles). For BAFF spiked into human serum, the yield of affinity purification was also about 10% of the initial already reduced activity, suggesting that serum-exposed affinity-purified BAFF in a 60-mer-favorable buffer does not regain extra activity (Fig. S3H, black circles and triangles). When the affinity purification was performed with a monoclonal anti-BAFF antibody (belimumab) that for steric hindrance reasons cannot bind BAFF 60-mer before it first dissociates into 3-mers [40], almost all BAFF 60-mer activity incubated at pH 8.2 was recovered in the unbound fraction, indicating that very little BAFF 60-mer dissociated at any time over the 3 days incubation period at pH 8.2 (Fig. S3E, white squares). Belimumab however depleted greater than 80% of BAFF 60-mer activity spiked into PBS or FCS, about 10% of which was recovered post elution (Fig. S3C, G, white triangles), suggesting that BAFF affinity-purified (as 3-mer) on the anti-BAFF antibody can subsequently re-associate as active 60-mer. However, for BAFF 60-mer spiked into human serum, affinity-purification on anti-BAFF gave the exact same results as affinity-purification on TACI-Fc, indicating again that 60-mer activity cannot be restored after exposure to human serum, even after serum removal by affinity-purification and buffer exchange in a 60-mer favourable buffer (Fig. S3H, I).

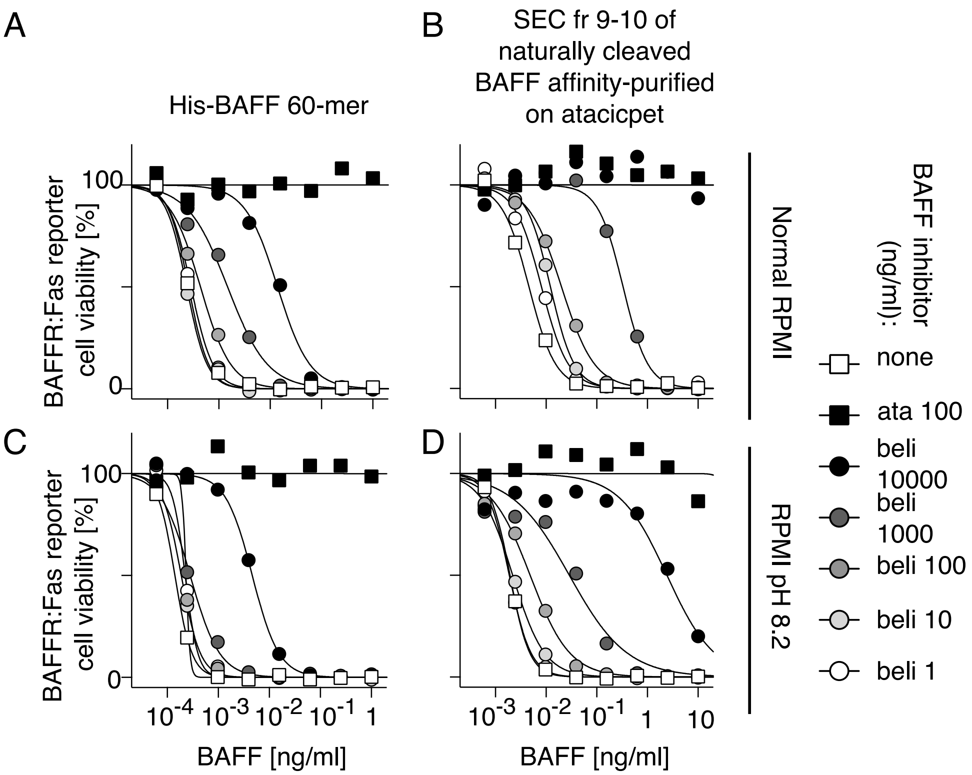

Supplementary Figure 4 (related to Figure 11). **Better detection of BAFF 60-mer by reporter cells grown at pH 8.2.**

(A) Titration of His-BAFF 60-mer on BAFFR:Fas reporter cells grown in RPMI 10% FCS in the presence of the indicated amounts of atacicept or belimumab. Cell viability was measured with the PMS/MTS test.

(B) Same as panel A, except that the high molecular weight fraction (SEC fr 9-10) of naturally cleaved BAFF 60-mer was used.

(C) Same as panel A, except that cells were grown in RPMI 10% FCS buffered at pH8.2 with 50 mM Hepes buffer.

(D) Same as panel B, but with cell grown at pH 8.2.

This experiment was performed once in this format.

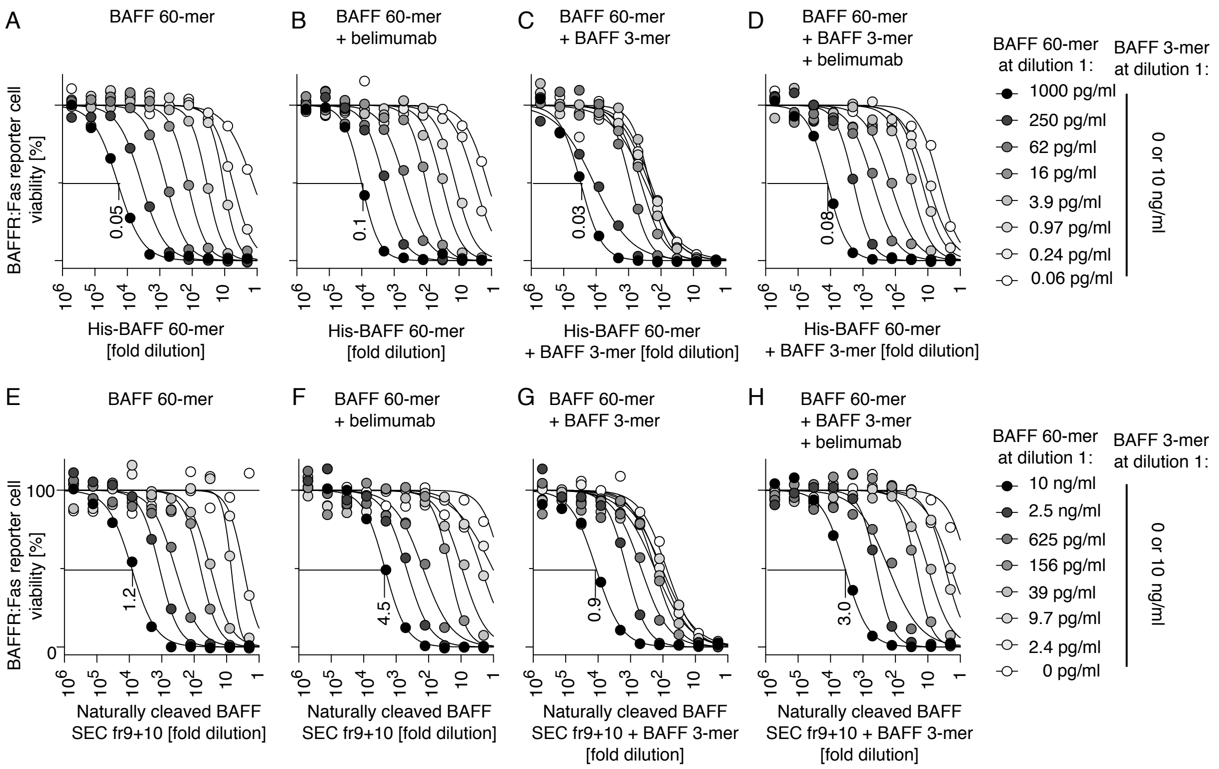

Supplementary Figure 5 (related to Figure 11). **Specific detection of BAFF 60-mer in the presence of BAFF 3-mer.**

(A) Titration of His-BAFF 60-mer on BAFFR:Fas reporter cells grown in RPMI 10% FCS pH8.2, starting at the indicated first concentration of BAFF 60-mer (before addition of cells). Cell viability was measured with the PMS/MTS test.

(B) Same as panel A, except that all wells contained belimumab at 100 ng/ml.

(C) Same as panel A, except that all wells contained Flag-BAFF 3-mer at 10 ng/ml.

(D) Same as panel A, except that all wells contained Flag-BAFF 3-mer at 10 ng/ml and belimumab at 100 ng/ml.

(E-H) Same as panels A-D, except that the high molecular weight fraction of naturally cleaved BAFF was used instead of His-BAFF 60-mer.

This experiment was performed once in this format.

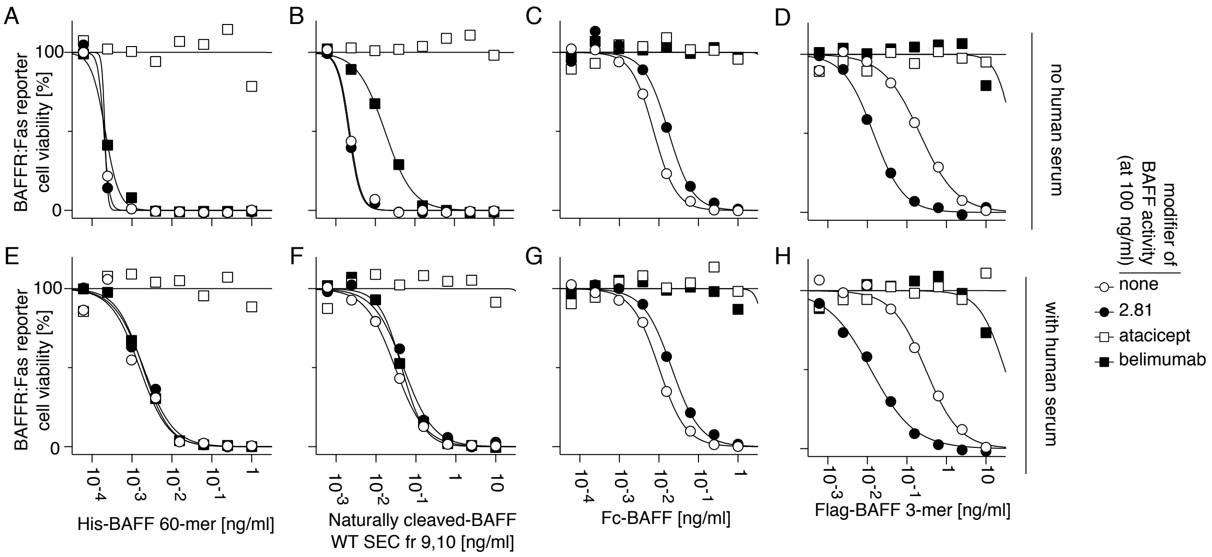

Supplementary Figure 6 (related to Figure 11). **Reporter cell-based assay for the detection of BAFF 60-mer versus BAFF 3-mer.**

(A) Titration of His-BAFF 60-mer on BAFFR:Fas reporter cells grown in RPMI 10% FCS pH8.2, in the presence or absence of modifiers of BAFF activity at 100 ng/ml (none: white circles, anti-BAFF 2.81: black circles, atacicept: white squares or belimumab: black squares). Cell viability was measured with the PMS/MTS test. **(B-D)** Same as panel A, except that the high molecular weight fraction of naturally cleaved BAFF **(B)**, Fc-BAFF **(C)** or Flag-BAFF 3-mer **(D)** were used.

(E-H) Same as panels A-D, except that 2 µl of normal human serum was added in each well and incubated for 5 min prior to addition of cells. This experiment of panels A and E was performed 3 times, B and F twice and the rest was performed once in this format.

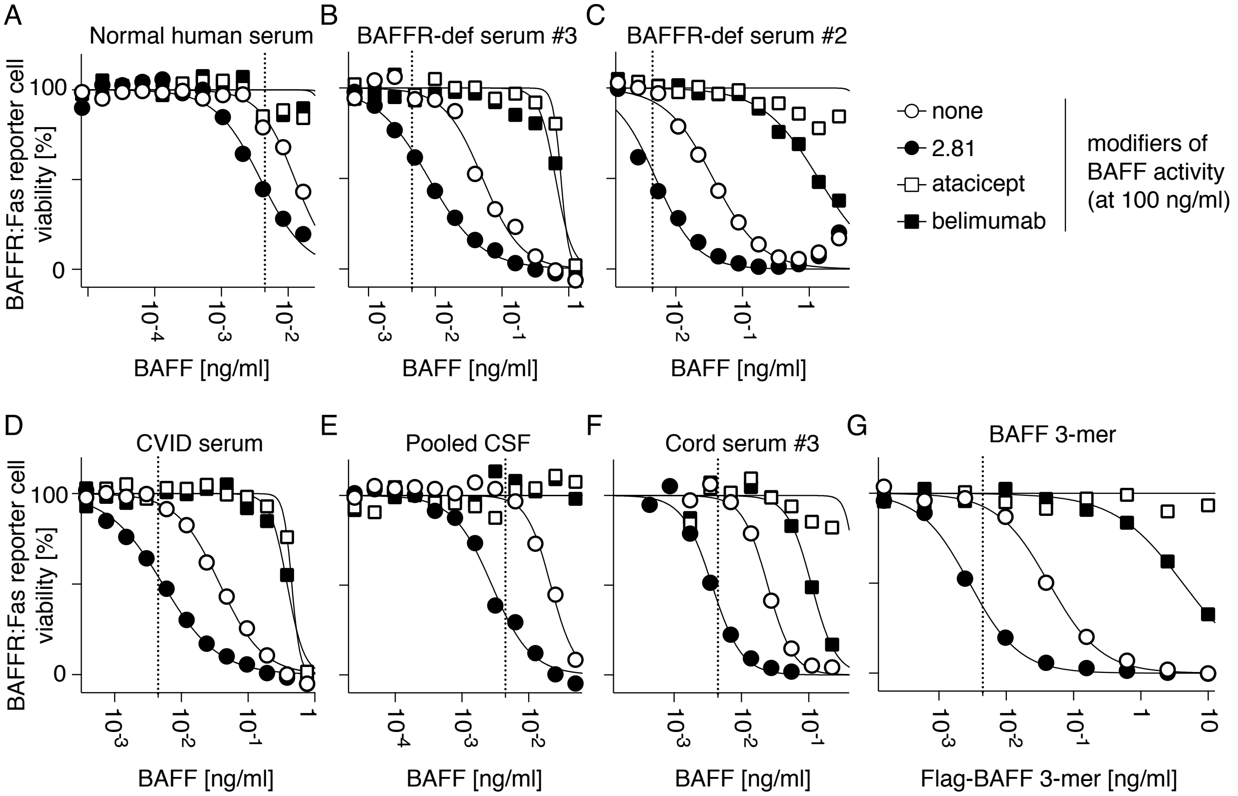

Supplementary Figure 7 (related to Figure 11). **Detectable BAFF 3-mer and 60-mer activities in a cord blood sample, but 3-mer only in CSF and adult human sera.**

A cell-based assay to distinguish BAFF 60-mer and BAFF 3-mer thanks to modifiers of activity was performed as described in the legend to Fig. 9, using serial dilutions of adult sera (25 µl in first well), of CSF (50 µl in first well) or of cord serum (10 µl in first well) that were quantified for BAFF by BAFF ELISA at neutral pH against a standard curve of the same Flag-BAFF 3-mer as that used in panel G. **(A)** Normal human serum. **(B-C)** Two independent serum samples from the same BAFFR-deficient person (identified as #2 and #3 in Fig. 1A). **(D)** Serum from a CVID patient. **(E)** Pooled, non-concentrated CSF samples from multiple sclerosis patients (4-7 in Fig. 7D, E). **(F)** Serum from cord blood (identified as #3 in Fig. 2F). **(G)** Recombinant Flag-BAFF 3-mer. The EC_{50} of reporter cell killing by BAFF in the presence of antibody 2.81 was calculated for each sample, including Flag-BAFF. The average of these values is shown in each graph as a vertical dotted line. Experiments in panels A-E and G were performed at least three times. Experiment in panel F was performed once because of limiting amounts of sample.

A cell-based assay detects BAFF 60-mer in the pg/ml range, even in the presence of an excess of BAFF 3-mer

A test able to identify BAFF 60-mer with high sensitivity in a small volume of sample, and without the cumbersome procedure of size-fractionation chromatography would be desirable. This test was based on BAFFR:Fas reporter cells because of their intrinsically high sensitivity to BAFF 60-mer. When BAFF 60-mer was titrated on BAFFR:Fas reporter cells, the marginal but existing and undesirable inhibitory activity of belimumab was reduced when culture medium was buffered at pH 8.2 compared to a parallel assay performed in conventional culture medium. At pH 8.2, the inhibitory activity of belimumab at 100 ng/ml on two different preparations of BAFF 60-mer was

considered negligible (Supplementary Fig. 4). Addition of an up to 10'000-fold excess of Flag-BAFF 3-mer to His-BAFF 60-mer prevented the interpretation of the results because both forms were active with the 3-mer masking the activity of low 60-mer concentrations. However, addition of belimumab in the assay specifically inhibited BAFF 3-mer activity, leaving the contribution of BAFF 60-mer almost intact (Supplementary Fig. 5A, B, C, D). These results also indicate that the specific activity of BAFF 3-mer is about 0.1% that of His-BAFF 60-mer, and that the assay can detect less than 1 pg/ml of His-BAFF 60-mer even in the presence of a vast excess of BAFF 3-mer. These results were confirmed with the 60-mer fraction of naturally cleaved BAFF, but in this case a) the specific activity of this 60-mer was lower than that of His-BAFF 60-mer (EC_{50} of ~1 versus ~ 0.1 pg/ml) and b) belimumab at 100 ng/ml slightly inhibited naturally cleaved BAFF 60-mer (EC_{50} of ~1 and 4 pg/ml without and with belimumab), suggesting that naturally cleaved BAFF could still partially dissociate into 3-mers under these conditions (Supplementary Fig. 3E, F, G, H). We have shown previously that the activity of mouse BAFF 3-mer can be increased by cross-linking with anti-mouse BAFF antibodies [41]. Here, we identified that the anti-hBAFF monoclonal antibody 2.81 [29] could similarly activate human Flag-BAFF 3-mer, but not His-BAFF 60-mer (Fig. 11A, E). This was also true for the 3-mer and 60-mer fractions of naturally cleaved wild type BAFF, and for the 3-mer fraction of BAFF with the mutation H218A that prevents 60-mer formation [39, 40] (Fig. 11B, C, F). Naturally cleaved BAFF with the mutation E223K that prevents both 60-mer formation and 3-mer to 3-mer interactions, and which is inactive on BAFFR:Fas reporter cells [40], regained high activity in the presence of anti-BAFF 2.81 (Fig. 11D). Thus, the analysis of BAFF activity on BAFFR:Fas reporter cells under four conditions allows distinguishing BAFF 60-mer from BAFF 3-mer. The four conditions are: a) sample alone, b) sample with the activating antibody 2.81 that activates BAFF 3-mer but not 60-mer, c) sample with BAFF inhibitory antibody belimumab that inhibits BAFF 3-mer much more efficiently than BAFF 60-mer and d) sample with atacicept that inhibits both BAFF 3-mer and 60-mer (Fig. 11A-F).
Exposure to human serum dissociates BAFF 60-mer into 3-mers (Fig. 5D), with concomitant loss of > 90% activity (Fig. 2A, B). We thus expected that serum should convert BAFF 60-mer into a BAFF with the properties of 3-mer in the reporter cell assay. Serum indeed reduced the activity of different BAFF 60-mers, but not that of Fc-BAFF oligomers or Flag-hBAFF 3-mer controls, yet the resulting activity of deactivated 60-mer were insensitive to inhibition by belimumab, while still sensitive to atacicept (Supplementary Fig. 6A-F). This was not due to serum-induced dysfunction of belimumab as serum did not alter its inhibitory effects on Flag-BAFF 3-mer (Supplementary Fig. 6G, H). Thus, the low activity of BAFF 60-mer incubated in serum is not or only minimally due to the formation of less active 3-mer, but reflects minute amounts of residual BAFF 60-mer, in line with the size-exclusion results of Fig. 4C.

Undetectable BAFF 60-mer activity in adult human sera and CSF samples using the reporter cell assay, but detectable 60-mer activity in a sample of cord blood.

Unmanipulated human sera and CSF were tested in the newly developed BAFFR:Fas reporter cell assay using various modifiers of BAFF activity. A sample of cord serum was also analysed, because we noticed that it contained some intrinsic activity on reporter cells (Fig. 2G, serum number 3). BAFF levels were measured by ELISA in these samples to allow quantitative comparisons. The activity of endogenous BAFF in all samples and that of recombinant Flag-BAFF 3-mer were enhanced by anti-BAFF 2.81, which is a characteristic of BAFF 3-mer (Supplementary Fig. 7A-G). Interestingly, EC_{50}s for killing reporter cells in the presence of mAb 2.81 were all in the same range (30 to 80 pg/ml), indicating that BAFF ELISA and BAFFR:Fas reporter cells are both suitable to

estimate total BAFF content in a sample. Belimumab inhibited BAFF activity in most samples, an indication for the absence of 60-mer (Supplementary Fig. 7A-E). In two instances, reporter cells were killed non-specifically at high serum concentration, regardless of the presence of belimumab or atacicept (Supplementary Fig. 7B, D). For the sample of BAFFR-deficient serum with the highest concentration of BAFF, inhibition with belimumab was less efficient than with atacicept, probably reflecting saturation of belimumab by BAFF as this was also seen with Flag-BAFF 3-mer at similar concentrations (Supplementary Fig. 7C and G). However, serum of cord blood could kill reporter cells in the presence of belimumab, but not atacicept, at BAFF concentrations that would have been inhibited by belimumab if it would have been BAFF 3-mer (Supplementary Fig. 7F). This suggests that this sample of cord blood contains detectable levels of BAFF 60-mer. This killing curve was actually comparable to that obtained with a mixture of 100 ng/ml of belimumab, 10 ng/ml of recombinant Flag-BAFF 3-mer and 40 pg/ml of recombinant BAFF 60-mer obtained from naturally cleaved BAFF (Supplementary Fig. 5H). To confirm the nature of this BAFF activity, the cord serum sample was size fractionated and analysed for BAFF protein content and activity. A small but distinct BAFF protein peak was detected in fraction 9, where BAFF 60-mer normally elutes, followed by a much more important peak of BAFF 3-mer (Fig. 8H). Signal in fraction 9 represented about 2% of total, which for a sample at 2 ng/ml amounts to 40 pg/ml, in good agreement with the concentration estimated by the activity test. Both peaks were active on reporter cells (Fig. 8H). Because this test was performed in the presence of the activating antibody 2.81, one expects the same specific activity for BAFF 3-mer and 60-mer. Fraction 9 contained 2.3% of the total BAFF activity, in good agreement with other estimations.

29. Kreuzaler, M., et al., Soluble BAFF levels inversely correlate with peripheral B cell numbers and the expression of BAFF receptors. J Immunol, 2012. 188(1): p. 497-503.
39. Cachero, T.G., et al., Formation of virus-like clusters is an intrinsic property of the tumor necrosis factor family member BAFF (B cell activating factor). Biochemistry, 2006. 45(7): p. 2006-13.
40. Vigolo, M., et al., A loop region of BAFF controls B cell survival and regulates recognition by different inhibitors. Nat Commun, 2018. 9(1): p. 1199.
41. Kowalczyk-Quintas, C., et al., Antibodies that block or activate mouse B cell activating factor of the tumor necrosis factor (TNF) family (BAFF), respectively, induce B cell depletion or B cell hyperplasia. J Biol Chem, 2016. 291(38): p. 19826-34.

Supplementary Table 1. Plasmids used in this study.

Plasmid	Designation	Protein encoded	Vector
ps336	Flag-hBAFF	HA signal-GPGQVQLQ hBAFF (137-285)	PCR3
ps544	hBAFF	hBAFF (aa 1-285)	PCR3
ps1377	pMSCS-puro	Modified pMSCV-puro (Clontech) with HindIII-BglII-EcoRI-NotI-XhoI-HpaI-ApaI sites	ps1377
ps2308	hBAFFR:Fas	HA signal-LE-hBAFFR (aa 2-71)-EFGSVD-hFas (aa 169-355)	ps1377
ps2309	hBCMA:Fas	HA signal-VQCEVKLVPRGS-hBCMA (aa 2-54)-VD-hFas (aa 169-355)	ps1377
ps2394	hBAFF H218A	hBAFF (aa 1-285), H218A	PCR3
ps2565	His-hBAFF	MRGSHHHHHHGS-h BAFF (aa134-285)	pQE
ps2825	Fc-hBAFF	HA signal LD h Fc (h IgG1 aa245-470) RSPQPQPKPQPKPEPEGSL h BAFF (aa136-285)	PCR3
ps3196	hBAFF E223K	hBAFF (aa 1-285), E223K	PCR3
ps3449	hBCMA-Fc	Modifiied Ig light chain signal-h BCMA (1-51)-VDHHHHHHLD-PreSci-h Fc (h IgG1 aa 245-470)	

Modified Ig light chain signal: METDTLLLWVLLLWVPGVHG. PreScission site (PreSci): LEVLFQGP.

Supplementary Table 2. Sample dilutions for BAFF ELISA post-size exclusion chromatography.

Fig.	Samples analyzed by SEC	Dilution or concentration of fractions	Vol. for ELISA
3A	50 µg of Flag-BAFF	dilution 1:50	10 µl
3C	100 µg of His-hBAFF 60-mer	dilution 1:50	5 µl
3E	Naturally cleaved BAFF in 10 ml supernatants, affinity purified on atacicept	none	1 µl
4A,C	40 ng His-hBAFF 60-mer in buffer or serum	none	66.6 µl
5C,D	160, 2.25 or 0.024 µg of His-hBAFF 60-mer in buffer or serum	dilution 1:490, 1:70 or none	70 µl
7A	200 µg of His-hBAFF 60-mer	dilution 1:12'500, then conc. 10-fold by lyophylization	50 µl
7C	200 µl of 16-fold concentrated pooled CSF	conc. 5-fold by lyophilization	5 or 50 µl
7E	200 µl of pooled CSF depleted with 104- or 5E1-Sepharose	conc. 10-fold by lyophilization	40 µl

7G	500 µl CSF affinity-purified on atacicept	conc. 10-fold by lyophilization	40 µl
7G	500 µl CVID serum affinity-purified on atacicept	conc. 10-fold by lyophilization	40 µl
8B-Q	200 µl of the indicated sera (excpt 8G, 100 µl)	conc. 10-fold by lyophilization	30 µl (8H: 40 µl)
10A	200 µl cord serum #11	none	150 µl
10C	(100 µl depleted cord serum #11) + (100 µl Hepes pH 8.2, 30 mM NaCl, 10 µg/ml BSA, 10 ng/ml naturally cleaved BAFF 60-mer fr 9+10)	none	150 µl
